# The pyrazolyl-urea GeGe3 inhibits tumor angiogenesis and reveals dystrophia myotonica protein kinase (DMPK)1 as a novel angiogenesis target

**DOI:** 10.18632/oncotarget.22598

**Published:** 2017-11-21

**Authors:** Elda Meta, Beat A. Imhof, Patricia Ropraz, Richard J. Fish, Chiara Brullo, Olga Bruno, Adama Sidibé

**Affiliations:** ^1^ Department of Pharmacy, Medicinal Chemistry Section, University of Genoa, 16132 Genoa, Italy; ^2^ Department of Pathology and Immunology, University of Geneva, 1211 Genève, Switzerland; ^3^ Department of Genetic Medicine and Development, University of Geneva, 1211 Genève, Switzerland

**Keywords:** angiogenesis, novel target, pyrazolyl-urea, kinase inhibitors, DMPK

## Abstract

The limitation of targeting VEGF/VEGFR2 signalling to stop angiogenesis in cancer therapy has been blamed on re-activation of alternative receptor tyrosine kinases by compensatory angiogenic factors. Targeting MAPK and PI3K signaling pathways in endothelial cells may be an alternative or complementary approach. Herein we aimed to evaluate the antitumor and antiangiogenic potential of a novel pyrazolyl-urea kinase inhibitor, GeGe3, and to identify its kinase targets.

We found GeGe3 to inhibit the proliferation of HUVEC and endothelial tube formation. GeGe3 impaired inter-segmental angiogenesis during development of zebrafish embryos. In mice, GeGe3 blocked angiogenesis and tumor growth in transplanted subcutaneous Lewis Lung Carcinomas. Screening for GeGe3-targeted kinases revealed Aurora B, Aurora C, NEK10, polo-like kinase (PLK)2, PLK3, DMPK1 and CAMK1 as candidate targets. Biochemical analysis of these kinases showed DMPK1 regulation upon VEGF challenge. Investigation of the role of DMPK1 in endothelial cells revealed DMPK1 as a novel mediator of angiogenesis that controls the activation of MAPK signaling, proliferation and migration. GeGe3 alters angiogenesis by targeting DMPK in tumor endothelial cells and pericytes.

The pyrazolyl-urea GeGe3, a novel blocker of MAPK and PI3K pathways, strongly inhibits physiological and tumor angiogenesis. We also report GeGe3-targeted kinase DMPK as a novel mediator of angiogenesis.

## INTRODUCTION

Angiogenesis, the formation of new blood vessels from existing vasculature, is indispensable for tumor growth and expansion beyond 2-mm^3^ in volume [1]. Tumor angiogenesis depends on angiogenic factors and the transduction of their signals in endothelial cells [2]. Extensive expression of Vascular Endothelial Growth Factor (VEGF)-A during tumor growth was reported for several tumor types [3, 4]. VEGF-A is the major angiogenic factor that activates VEGF receptors (VEGFR)-1 and -2 on endothelial cells. VEGFR activation triggers angiogenic migration, proliferation and survival of endothelial cells. Targeting the ligand, VEGF, with humanized neutralizing monoclonal antibody, or the receptors with receptor tyrosine kinase inhibitors, are promising cancer therapy approaches [5–8]. However, problematically, tumors express compensatory angiogenic factors to overcome VEGF blockade, which frequently leads to rebound in tumor angiogenesis.

Many compensatory and complementary angiogenic factors have been described in several tumor types. They include members of the VEGF family such as placental growth factors (PlGF), VEGF-C, VEGF structural homologs such as platelet-derived growth factor (PDGF), multifunctional growth factors such as basic fibroblast growth factor (bFGF), angiopoietin (Ang)-2, and chemokines such as interleukin (IL)-8 [9–11]. These factors signal through their specific receptors on endothelial and perivascular cells to support angiogenesis. Several neutralizing antibodies or tyrosine kinase inhibitors targeting the signal transduction of the compensatory factors are in development and some already went into clinical trials, alone or in combination with chemotherapy [12, 13]. Many of these strategies were as effective as blocking VEGF. Interestingly the tyrosine kinase inhibitors, used to block the signaling of compensatory factors, simultaneously also target downstream VEGF signaling. This suggests that targeting more common signaling pathways, rather than a specific factor, may be important for improving antiangiogenic therapy of tumors.

Serine/threonine kinases of the mitogen-activated protein kinases (MAPKs) such as p38-MAPK, extracellular response kinases (ERK) and mediators of the phosphoinositide 3-kinase (PI3K) signaling pathway such as protein kinase B (PKB/AKT) play paramount roles in tumor growth and angiogenesis [14, 15]. Indeed, angiogenic factors, including VEGF, bFGF, PlGF and IL-8 activate MAPKs and PI3K to induce migration, proliferation and survival of the endothelial cells that are involved in angiogenesis [16–20]. However serine/threonine kinases in the pathways of MAPK and PI3K have not been a main focus of antiangiogenic therapies, presumably because of their extended implications beyond pathological tissues. Exceptionally, Sorafenib (Nexavar^®^), which is a dual inhibitor of serine/threonine- (Raf/MEK/ERK) and tyrosine (VEGFR and PDGFR) kinases, was approved by the US Food and Drug Administration for the treatment of metastatic renal cell carcinoma and unresectable hepatocellular carcinoma [21–23]. Therefore, there is a therapeutic imperative to develop more serine/threonine kinase inhibitors that specifically interfere with tumor angiogenesis with minor toxicity for healthy tissues.

In this regard, the development of serine/threonine kinase-inhibiting pyrazole derivatives, with anti-cancer and antiangiogenic properties, is in expansion. Some were developed and described to present antitumorigenic and antiangiogenic potentials. They encompass diaminopyrazoles, trisubstituted pyrazoles, pyrazolo-benzodiazepines, and pyrazolo-pyrimidines [24–30]. In previous studies, we developed several chemical libraries that block activation of ERK1/2, p38MAPK and AKT in neutrophils, stimulated by IL-8 or formyl-methyl-leucyl-phenylalanine peptide and that inhibit neutrophil migration [31–36]. More recently we designed and synthesized a series of 27 pyrazolyl-ureas and imidazo-pyrazole-carboxamides and found them to differentially modulate the activity of ERK1/2, p38MAPK and AKT in VEGF-stimulated human umbilical vein endothelial cells (HUVECs) [37]. Our library of compounds revealed the ethyl 1-(2-hydroxypentyl)-5-(3-(3-(trifluoromethyl)phenyl)ureido)-1*H*-pyrazole-4-carboxylate (named here GeGe3) to be a potent inhibitor of HUVEC migration. This suggested that GeGe3 may be an angiogenesis inhibitor.

In this study, we characterize the activity of GeGe3 by evaluating its anti-angiogenic potential in physiological as well as pathological models of angiogenesis and identifying its putative molecular targets during this process.

## RESULTS

### The pyrazolyl-urea GeGe3 inhibits endothelial cell proliferation

**Figure 1:**
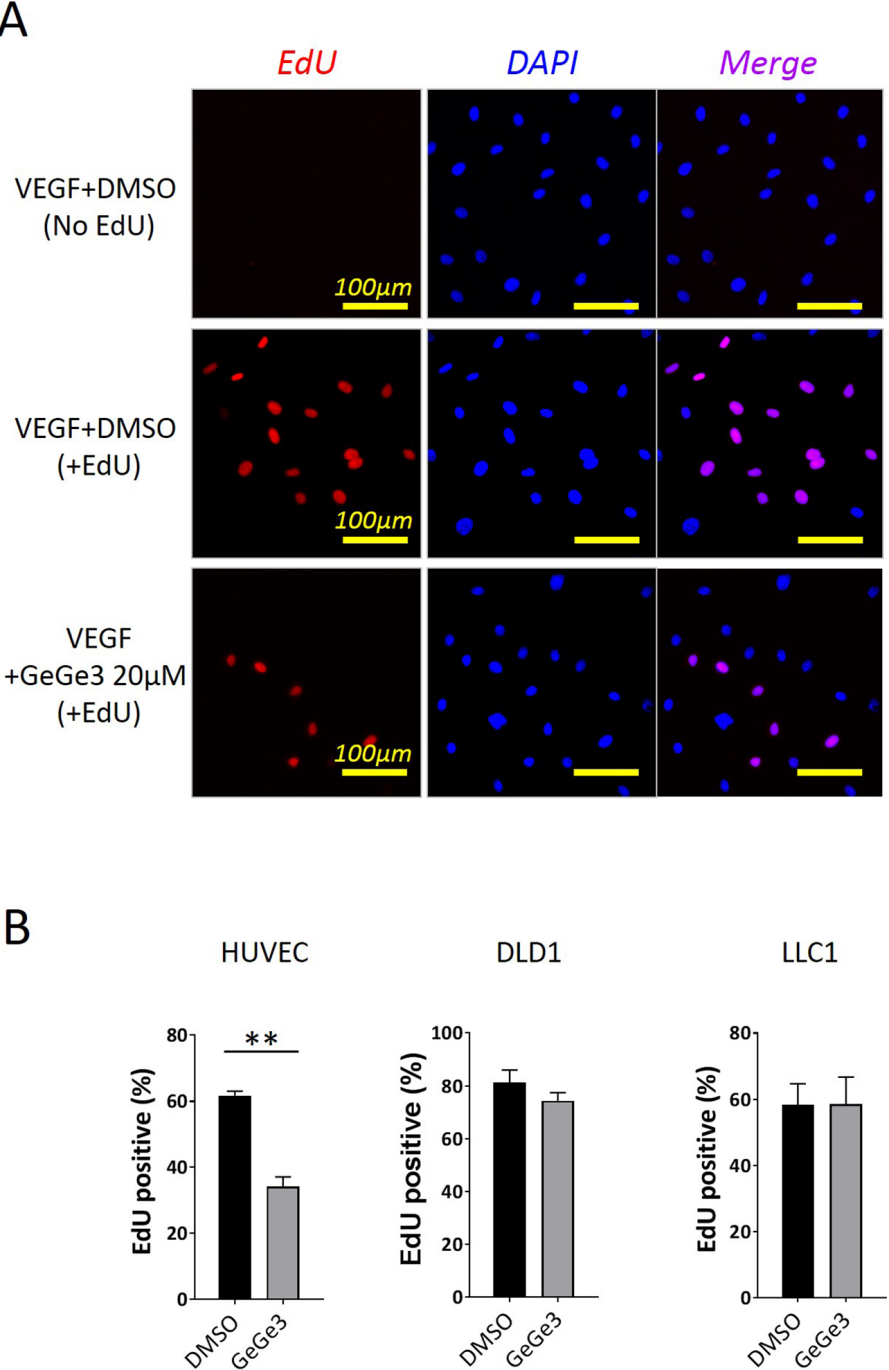
Effect of GeGe3 on endothelial cell proliferation and survival **(A)** Fluorescence imaging of proliferating endothelial cells in an EdU proliferation assay. Sparse endothelial cells were cultured with GeGe3 (20 μM) or DMSO and without (top) or with EdU (middle and bottom), for 20 hours. EdU incorporation is a marker of cell proliferation and was specifically detected by fluorescence microscopy after staining with the Click-iT kit according to the manufacturer (Thermo Fisher Scientific). Cell nuclei were stained with DAPI and the DAPI signal served to normalize between conditions. **(B)** Effect of GeGe3 on the proliferation of HUVECs, human colorectal adenocarcinoma and mouse Lewis Lung carcinoma cells. The percentages of EdU-positive nuclei are shown. Incubation of endothelial cell with GeGe3 significantly inhibited the proliferation of endothelial cells. Data are presented as mean ± sem. ^**^:p-value<0.001. Data were pooled from 3 independent experiments.

We recently demonstrated that VEGF-induced HUVEC migration was inhibited by GeGe3, a pyrazolyl-urea compound that we also found to block the activation of MAPK and PI3K signaling pathways in HUVEC [37]. Given the role of MAPK and PI3K signaling pathways in endothelial cell proliferation, our results suggested a potential capacity of GeGe3 to interfere with endothelial cell proliferation. We therefore examined GeGe3's inhibitory action on VEGF-induced proliferation of HUVECs. Proliferating cells were identified by their ability to incorporate a thymidine analogue, the 5-Ethynyl-2'-deoxyuridine (EdU), during cell division. We cultured HUVECs overnight with medium containing EdU, VEGF and GeGe3 or DMSO. As shown in Figure 1A, EdU signals were detected in nuclei, identified by DAPI staining, only when EdU was added to the medium demonstrating the functionality of the assay. The percentage of EdU+ nuclei decreased in the presence of GeGe3 (Figure 1B).

We tested whether GeGe3 would induce apoptosis of non-proliferating HUVECs as a general test of toxicity for cell survival. We analyzed the effects of overnight GeGe3 treatment on HUVEC membrane integrity as this is an indicator of cell apoptosis. Membrane integrity was examined by flow cytometry analysis of propidium iodide uptake (Supplementary Figure 2A). Treatment with GeGe3 did not increase the number of cell positive for propidium iodide (Supplementary Figure 2B). Consistently, HUVEC morphology was similar with and without GeGe3 demonstrating no effect of the compound on apoptosis of non-proliferating HUVECs (Supplementary Figure 2B).

We next tested whether GeGe3 could halt proliferation of other cell types, namely human colorectal adenocarcinoma (DLD1) and mouse Lewis lung carcinoma (LLC)-1 cell lines and found no effect of the compound (Figure 1B). Altogether, these results demonstrated GeGe3's inhibitory effect on endothelial cell proliferation but do not affect non-proliferating cell viability.

### GeGe3 inhibited physiological angiogenesis during development of zebrafish embryos

**Figure 2:**
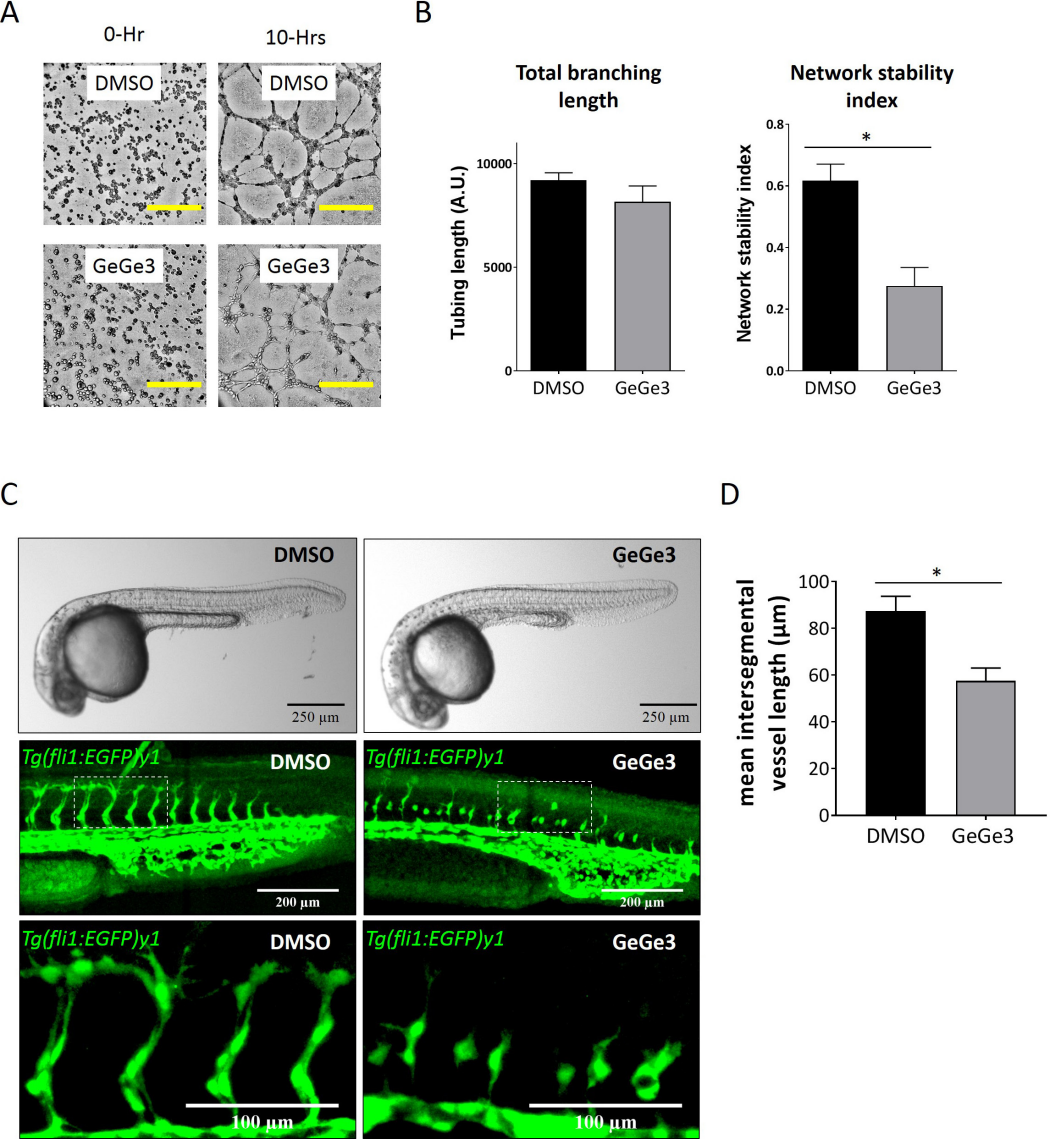
GeGe3 impaired tube formation *in vitro* and intersegmental angiogenesis of Tg(fli1:EGFP)y1 zebrafish embryos *in vivo* **(A)** Bright-field time-lapse imaging of HUVEC tube formation in the presence of GeGe3. An endothelial tube formation assay was performed in Geltrex™ matrix with VEGF/DMSO or VEGF/GeGe3 for 10 hours. Pictures are shown for the initial cells (0 hours) and after formation of tube-like structures (10 hours). Scale bar = 100μm. **(B)** Quantification of the effect of GeGe3 on angiogenesis *in vitro*. The total tube length was determined as well as the network stability index, which is the ratio of total tube length to the number of isolated blocks of segments. Data are presented as mean ± sem. ^*^:p-value<0.05. Data are representative of 4 independent experiments. **(C)** Images of whole 1 day post fertilization (dpf) zebrafish embryos after 8 hours of DMSO or GeGe3 treatment were acquired with a stereomicroscope. Scale bar = 250μm; middle panel: maximal projection of stack fluorescence images of zebrafish tail intersegmental vessels acquired with a confocal microscope, scale bar = 200μm; bottom panel: zoomed fluorescence images of intersegmental blood vessels, scale bar = 100μm. **(D)** Quantification of GeGe3's effect on intersegment angiogenesis. The mean length of the intersegmental blood vessels was measured and compared between GeGe3 and DMSO. Data are presented as mean ± sem; ^*^:p-value<0.05. Data are representative of three independent experiments with at least five fish embryos per group per experiment.

Next we investigated the antiangiogenic potential of GeGe3 with an endothelial tube formation assay in polymerized matrix. This is a classic *in vitro* angiogenesis test that recapitulates major events occurring during angiogenesis, including endothelial cell sprouting, migration and connection. Geltrex™ matrices were polymerized to form a solid support. We added HUVECs onto polymerized Geltrex™ matrix in medium containing VEGF and either GeGe3 or DMSO before imaging for 10 hours. As shown in Figure 2A, endothelial cells under both conditions rearranged to form tube-like structures and formed a network. The total length of the tubes was determined and similar lengths were found for GeGe3 treatment and controls (Figure 2B). We also determined the network stability index, which was calculated as the ratio of total tube length to total counts of isolated blocks. Interestingly, the network stability index showed that GeGe3 inhibits the capacity of HUVECs to form stable networks.

The inhibition of endothelial cell migration shown in our previous study and the blockade of endothelial cell proliferation and stable tube formation demonstrated in this work support the potential interest of GeGe3 for targeting angiogenesis *in vivo*. We therefore evaluated the antiangiogenic features of GeGe3 on physiological angiogenesis of developing *Tg(fli1:EGFP)y1* zebrafish embryos, which express the enhanced green fluorescent protein (EGFP) in endothelial cells, convenient for imaging. 1 day-post-fertilization zebrafish embryos were incubated in E3 medium containing either GeGe3 or DMSO for eight hours. The embryos were photographed and the effect of GeGe3 on vessel formation was analysed. As shown in Figure 2C and 2D, the angiogenic, intersegmental vessels of GeGe3-treated embryos were significantly shorter compared to DMSO-treated embryos and demonstrated poor overall morphology including incomplete sprouting at somite boundaries. These results demonstrated that GeGe3 impaired intersegmental vessel angiogenesis during development. Altogether, these results demonstrate that GeGe3 is a potent blocker of angiogenesis *in vitro* and *in vivo*.

### ERK and AKT are not primary targets of GeGe3

**Figure 3:**
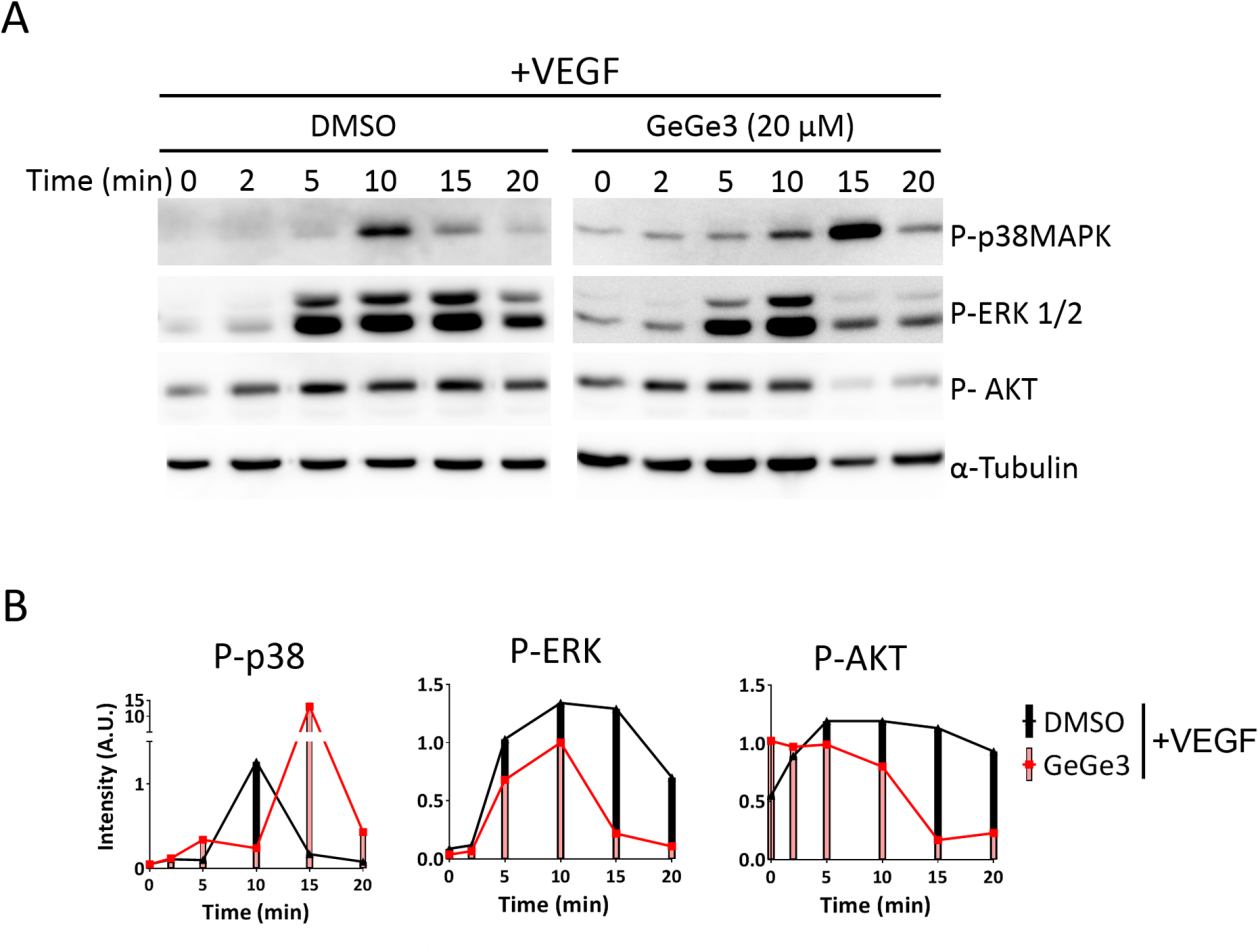
Effect of GeGe3 on VEGF-induced activation of p38MAPK, P-ERK and AKT **(A)** Kinetic effect of GeGe3 on VEGF-induced activation of p38MAPK, ERK and AKT. Confluent HUVECs were starved for at least 4 h. The cells were then incubated for 10-min with fresh medium containing GeGe3 or control DMSO and then VEGF (50 ng/ml) was added for the different time periods shown. Finally protein extracts were analyzed for phosphorylation of p38MAPK, ERK1/2 and AKT by Western blotting. α-tubulin levels were used to control protein loading and for normalization. **(B)** Time-course quantification of normalized phosphorylated p38MAPK, ERK and AKT. Data are representative of 2 independent kinetic experiments.

The capacity of GeGe3 to block physiological angiogenesis *in vivo* led us to investigate its direct targets in endothelial cells. Previously we showed that GeGe3 amplified VEGF-induced activation of p38MAPK but conversely blocked that of ERK1/2 and AKT, after VEGF-stimulation of HUVECs [37]. However, it was unknown whether these kinases were the direct targets of GeGe3. We then investigated the kinetic inhibitory profile of GeGe3 action on MAPK and PI3K signaling pathways. Therefore we analyzed the phosphorylation of p38MAPK, ERK1/2 and AKT over time during VEGF stimulation. Confluent HUVECs were starved for >4 hours to synchronize cell cycling and reduce baseline phosphorylation levels. Then the cells were incubated for 10-min with fresh medium containing GeGe3 or DMSO to maximize inhibition of the compound targets before VEGF stimulation. Next we stimulated the cells with VEGF (50 ng/ml) in presence of GeGe3 or control DMSO for different times (0, 2, 5, 10, 15 and 20-min). The protein extracts were analyzed for phosphorylation of p38MAPK, ERK1/2 and AKT by Western blotting normalized to α-tubulin levels (Figure 3A and 3B). Indeed, HUVEC stimulations by VEGF or GeGe3 during short time periods (0 to 20-min) showed no effect on protein contents of the investigated kinases (Supplementary Figure 3). Thus we used housekeeping proteins such as α-tubulin or β-actin for protein loading normalization throughout this study. We noticed two phases of GeGe3 action on VEGF-induced activation of the three proteins. During the early phase, up to 10-min of VEGF stimulation, p38MAPK was not activated by VEGF in the presence of GeGe3. Interestingly, in the second phase, the presence of GeGe3 along with VEGF led to a rebound of p38MAPK activation at 15-min with a higher amplitude than the control. This delay induced by GeGe3 on p38MAPK activation suggested that it may act upstream of p38MAPK activation and therefore interfere with its phosphorylation in the early period. This inhibitory effect might be alleviated in the second period through p38MAPK activation by alternative upstream mediators.

A different profile was found for GeGe3 action on ERK and AKT activation in response to VEGF. During the early period GeGe3 slightly decreased activation of ERK and AKT. The second phase was characterized by a dramatic inhibition of their activation by VEGF. These results indicate that GeGe3 does not interfere with the upstream kinases involved in the initial activation of ERK and AKT. However, GeGe3 may target serine/threonine kinases required for signal amplification and/or maintenance of ERK and AKT activation at a later time after VEGF stimulation. Altogether, these results suggest that p38MAPK, ERK and AKT are not the primary targets of GeGe3.

### VEGF induces DMPK phosphorylation, which is a direct target of GeGe3 in endothelial cells

**Figure 4:**
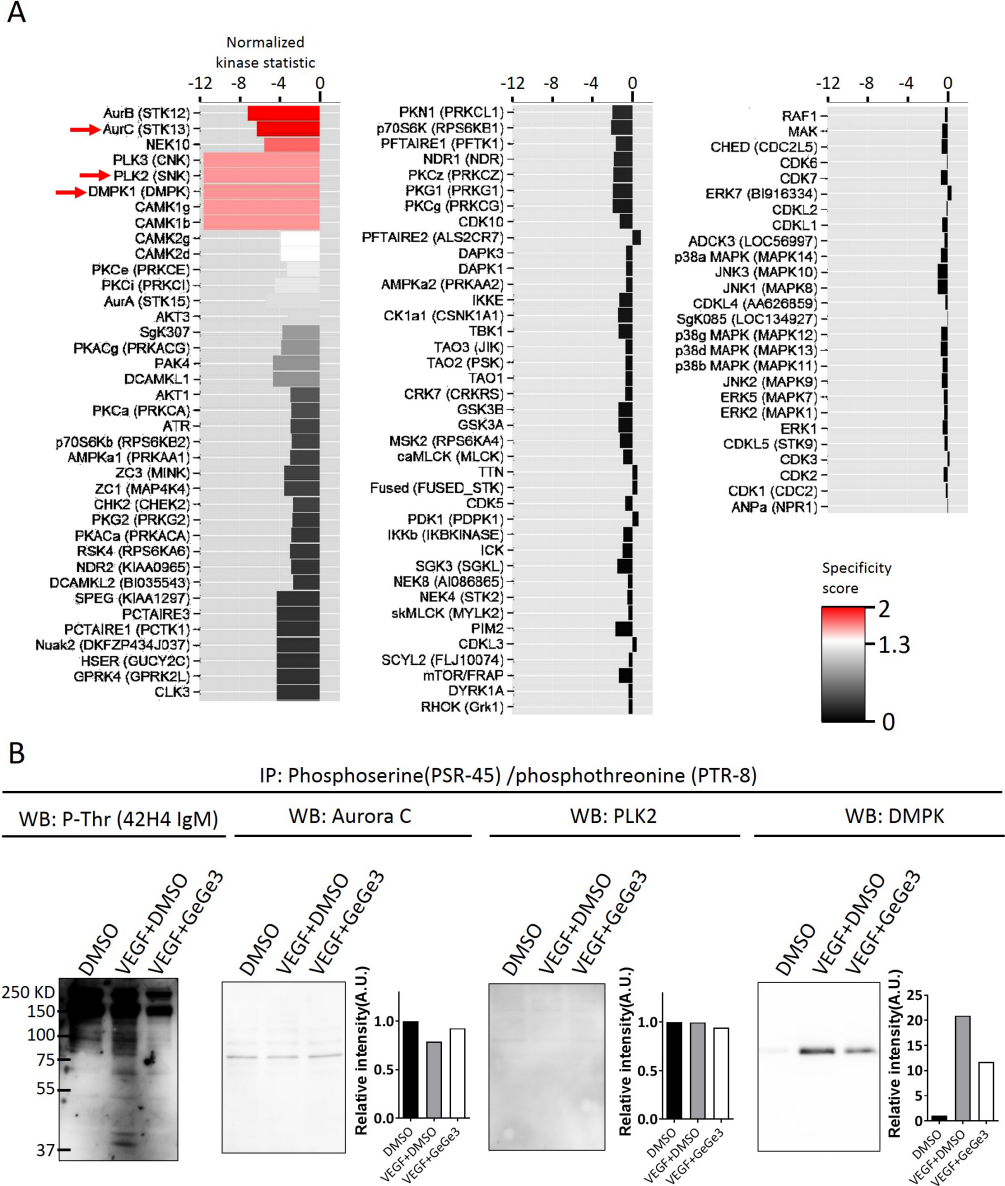
The serine/threonine kinase targets of GeGe3 in endothelial cells **(A)** Serine/threonine kinase array showing differences of kinase activity between DMSO and GeGe3. Total proteins were extracted from endothelial cells and the effect of GeGe3 was directly tested on the activity of specific serine/threonine kinases with a PamStation 12^®^ instrument. Data were analyzed with Bionavigator software (PamGene) and presented as fold-difference from the DMSO control (Normalized kinase statistic). Negative values indicate inhibition and positive values activation. The specificity score of the effect of GeGe3 is color-coded (black: non-specific and red: highly specific). The 102 first kinases are shown. **(B)** VEGF induction of candidate kinases targeted by GeGe3 in endothelial cells. Confluent HUVECs were starved for at least 4 hours. Next the cells were incubated for 10 min with fresh medium containing GeGe3 (20 μM) or control DMSO and then VEGF (50 ng/ml) was added for 5-min before protein extraction. Serine/threonine phosphorylated proteins were immunoprecipitated with combination of anti-phosphothreonine (PTR-8) and antiphosphoserine (PSR-45) antibodies. The precipitates were analyzed for phospho-threonine with a different phospho-specific antibody (42H4 IgM) to validate the precipitation. The presence of Aurora C, PLK2 and DMPK1 in the phospho-precipitates was determined with specific antibodies against indicated total proteins. B and intensity level is indicative of the phosphorylated form of the proteins as the immunoprecipitates of phosphothreonine/phosphoserine proteins were used for blotting. Data are representative of three independent experiments.

Next we aimed at unveiling the serine/threonine kinases that are directly targeted by GeGe3 in endothelial cells during VEGF stimulation. Therefore we performed a multiplex serine/threonine kinase assay *in vitro* by using a PamChips array on a PamStation^®^12 instrument. These arrays have immobilized peptides and combinatorial phosphorylation of these peptides allows identification of specific kinases by bioinformatics. In our experiment the kinase source was the lysates of confluent HUVEC stimulated with VEGF. The kinase assay was performed in presence of GeGe3 or DMSO, and the serine/threonine kinase activities are reported in Figure 4A. The data from this assay confirmed that p38MAPK, ERK and AKT were not the primary targets for GeGe3. Instead the compound strongly inhibited other kinases, namely Aurora B, Aurora C, NEK 10 (never-in-mitosis A type 10, NIMA or NEK), PLK (polo like kinases)2, PLK3, DMPK (Dystrophia Myotonica Protein Kinase)1 and CaMK1 (Calcium/calmodulin-dependent protein kinase type 1).

We focused on three GeGe3-targeting kinases from this short list, namely Aurora C, PLK2 and DMPK1. The selection of Aurora C and PLK2 was based on their known involvement in endothelial cell proliferation and angiogenesis [39, 40]. We also studied DMPK1 because it remains unexplored in endothelial cells and plays a crucial role in calcium homeostasis in other cells such as neurons and muscle cells [41, 42]. Starved, confluent HUVECs were incubated for 10-min with medium containing GeGe3 or control DMSO and then VEGF was added afterwards for 5-min to explore early signaling events. Proteins that were phosphorylated on serine/threonine residues were immunoprecipitated with a combination of two antibodies against phosphoserine and phosphothreonine in each sample. The immuno-precipitates were analyzed by western blotting for the presence of the candidate kinases. As shown in Figure 4B, within the precipitates, we detected phosphothreonine proteins with another anti-phosphothreonine to validate the precipitations. Blotting of Aurora C of the precipitates showed a small band that was similar in all three conditions (Figure 4B). PLK2 also was barely detectable in the precipitates of any condition. Interestingly, a small band of DMPK1 was precipitated with phosphoserine/phosphothreonine antibodies in non-stimulated HUVECs. The amount of phosphoDMPK1 was clearly increased by VEGF stimulation but this induction was inhibited by GeGe3. This demonstrated that among the three kinases analyzed, only DMPK1 was activated very early by VEGF stimulation in endothelial cells. Altogether, these results demonstrate that GeGe3 directly de-activates a small number of serine/threonine kinases upon VEGF stimulation of endothelial cells.

### The decrease of DMPK1 protein inhibits endothelial cell proliferation, migration and tube formation *in vitro*

**Figure 5:**
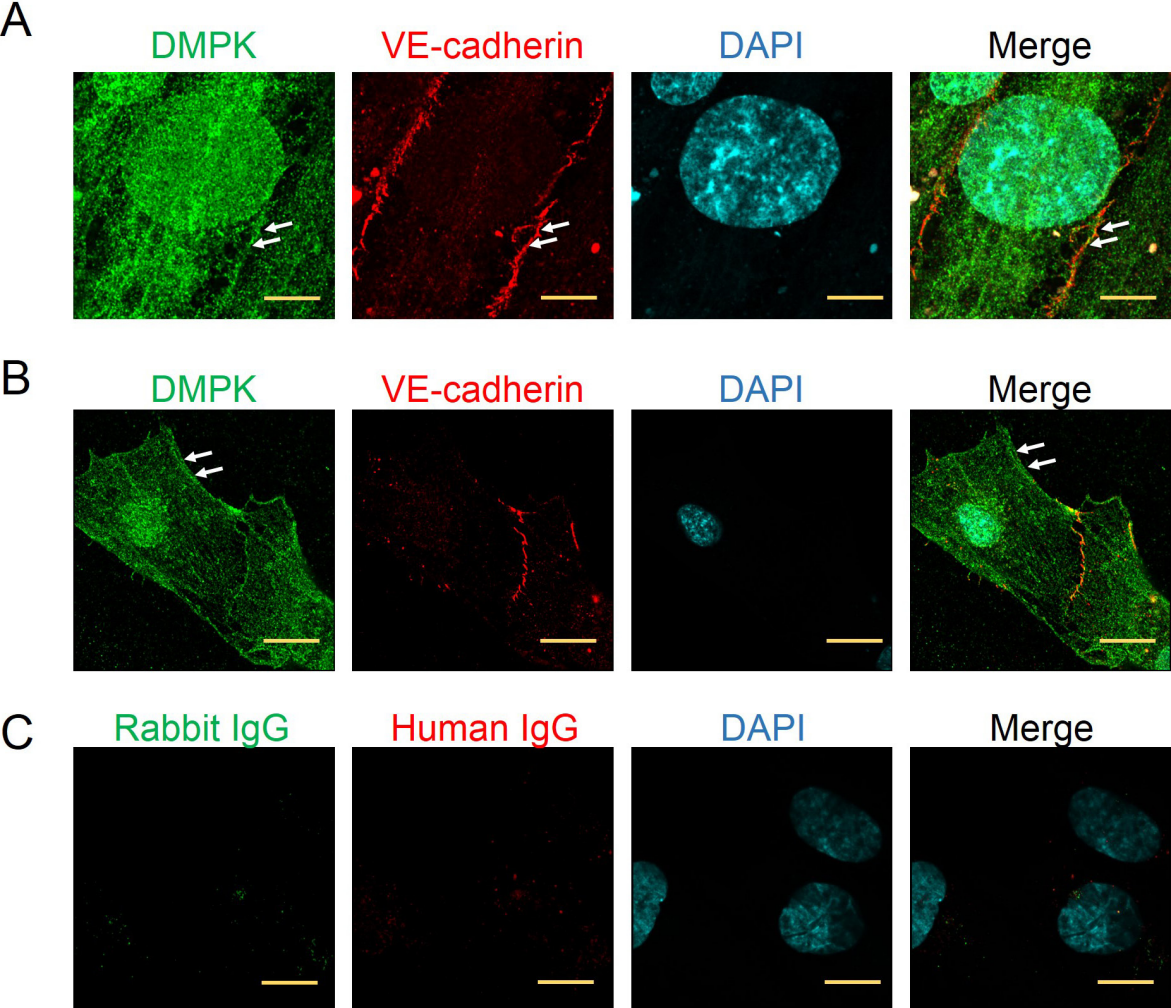
Localization of DMPK in confluent and migrating endothelial cells **(A)** Super-resolution confocal imaging of DMPK localization in endothelial cells at confluence. Scale bar = 5μm, arrows indicate ‘junctional’ localization of DMPK. **(B)** Super-resolution confocal imaging of DMPK localization in migrating endothelial cells. Scale bar = 10 μm. Arrows indicate DMPK localization at the cell edge. HUVEC junctions were stained with a recombinant human anti-VE-cadherin and the nuclei with DAPI. **(C)** Negative control staining of HUVEC. Rabbit IgG and human IgG were used as negative controls and showed no staining. Scale bar = 5 μm.

DMPKs are homologous to the Rho family serine/threonine kinases. DMPK1 is involved in calcium homeostasis and has mainly been studied for its functions in muscle and brain and it has not been studied in endothelium. As we observed rapid activation of DMPK1 in endothelial cells, after VEGF stimulation, and the direct targeting of this kinase by GeGe3, we hypothesized a role for DMPK1 in endothelial cells and angiogenesis. We explored DMPK1 distribution in endothelial cells by confocal microscopy. In confluent HUVECs, DMPK1 was present in the nucleus and in the cytoplasm, in structures resembling the endoplasmic reticulum. We also saw DMPK1 at cell junctions where it colocalized with VE-cadherin (Figure 5A). DMPK1 also accumulated at endothelial cell borders in sparse cells (Figure 5B). The results above have shown the expression of DMPK1 by endothelial cells, its localization suggesting a role in endothelial cell movement and its activation by VEGF that was blocked by GeGe3.

**Figure 6:**
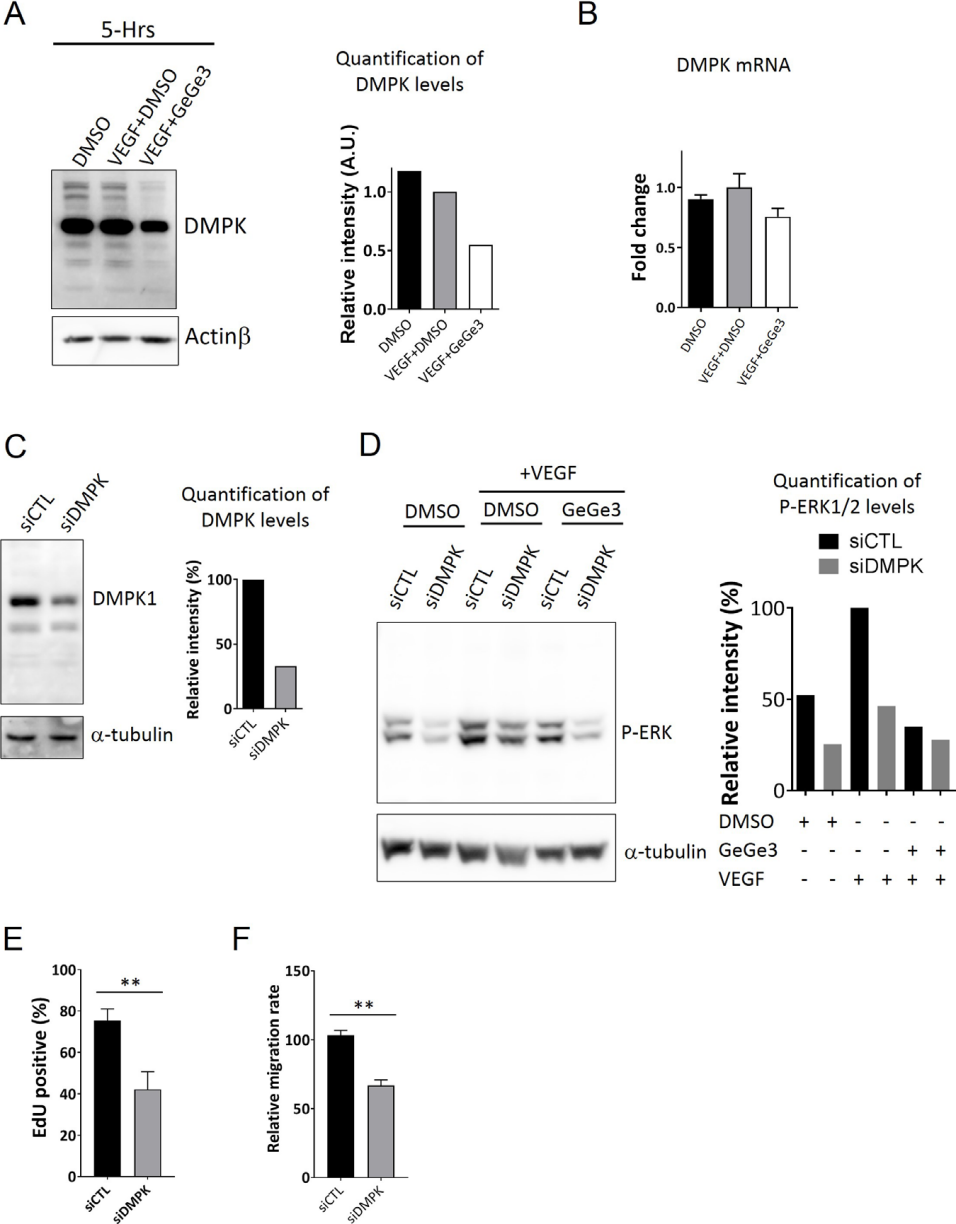
Downregulation of DMPK protein by GeGe3 impairs HUVEC proliferation and migration **(A)** Effect of GeGe3 on DMPK protein level in endothelial cells. Confluent HUVECs were incubated with VEGF (50 ng/ml)/GeGe3 (20μM) or VEGF/DMSO 5 hours before protein extraction. Protein extracts were analyzed by western blotting for DMPK and actinβ (left). DMPK band intensity was normalized with actinβ and plotted as relative to VEGF+DMSO (right). DMPK protein was decreased after 5 hours of endothelial cell treatment with GeGe3. **(B)** Effect of GeGe3 on DMPK mRNA expression in endothelial cells. HUVEC were treated as described in (A) before total RNA extraction and analysis of DMPK mRNA levels in each condition by qPCR. DMPK mRNA levels are presented as mean fold-difference to the VEGF+DMSO condition. **(C)** Western blot analysis of DMPK expression 48 hours after its knockdown by siRNA (left) and its quantification (right). **(D)** Analysis of DMPK crosstalk with MAPK signaling pathways. On the left is a western blot of phospho-ERK1/2 and actin from control or siDMPK-transfected HUVECs. These cells were stimulated as indicated: with DMSO alone, with VEGF+DMSO or VEGF+GeGe3; on the right is the relative quantification of ERK1/2 phosphorylation on the presented blots. **(E)** Effect of DMPK knockdown on HUVEC proliferation and **(F)** Effect of DMPK knockdown on HUVEC migration. Data are presented as mean ± sem. ^*^:p-value<0.05. The results are representative of 2-3 independent experiments.

Next we tested possible effects of long term GeGe3-treatment of endothelial cells on DMPK1 protein. Thus endothelial cells were treated with VEGF+GeGe3 or VEGF+DMSO for 5 hours and the protein extract were analyzed for DMPK1 levels by western blotting. Interestingly, long term treatment of HUVEC with GeGe3 decreased the DMPK1 protein level (Figure 6A). GeGe3 clearly interferes with both the kinase activity and the expression of DMPK1. Therefore DMPK1 kinase activity may be important for its stability or it may feedback to maintain its own expression. However, GeGe3 did not decrease DMPK1 mRNA (Figure 6B). We then favor the hypothesis that DMPK1 stability depends on its kinase activity in endothelial cells.

Next we investigated whether the decrease in DMPK1 protein induced by GeGe3 could account for its anti-proliferative and antiangiogenic effect. To test this, we downregulated DMPK1 for 72 hours in HUVECs by RNA interference (Figure 6C). The protein level of DMPK1 was reduced to ~30% in siDMPK1 compared to control cells. With these transfected cells, we first tested whether the blockade of MAPK by GeGe3 could be due to a direct action on DMPK1. Analysis of ERK1/2 phosphorylation showed that VEGF activated less MAPK signaling pathway in siDMPK1 cells compared to control cells (Figure 6D). However, DMPK knockdown did not alter ERK phosphorylation as much as GeGe3 treatment. Nonetheless DMPK1 downregulation significantly reduced proliferation and migration of endothelial cells (Figure 6E, 6F). As DMPK1 is a regulator of calcium homeostasis, we investigated whether the calcium-dependent CaMK1 could be a mediator of DMPK1 action on MAPK signaling. Therefore, we analyzed the status of CaMK1 activation in HUVECs stimulated by VEGF (50 ng/ml) for 5-min in presence of GeGe3 (Supplementary Figure 4). We used a phospho-specific antibody against phospho-Thr177-CaMK1, which indicates the extent of CaMK1 activation. VEGF stimulation did not induce any change in CaMK1 activation. This indicates that DMPK1 action is not mediated through CaMK1 activation at least during early time periods. Altogether these results demonstrate that VEGF-induced endothelial cell proliferation and migration are influenced by DMPK1 and this could explain how GeGe3 interferes with angiogenesis. However, the calcium-dependent CaMK1 does not seem to be the mediator that remains to be clearly identified.

### GeGe3 targets tumor angiogenesis and decreases tumor growth

**Figure 7:**
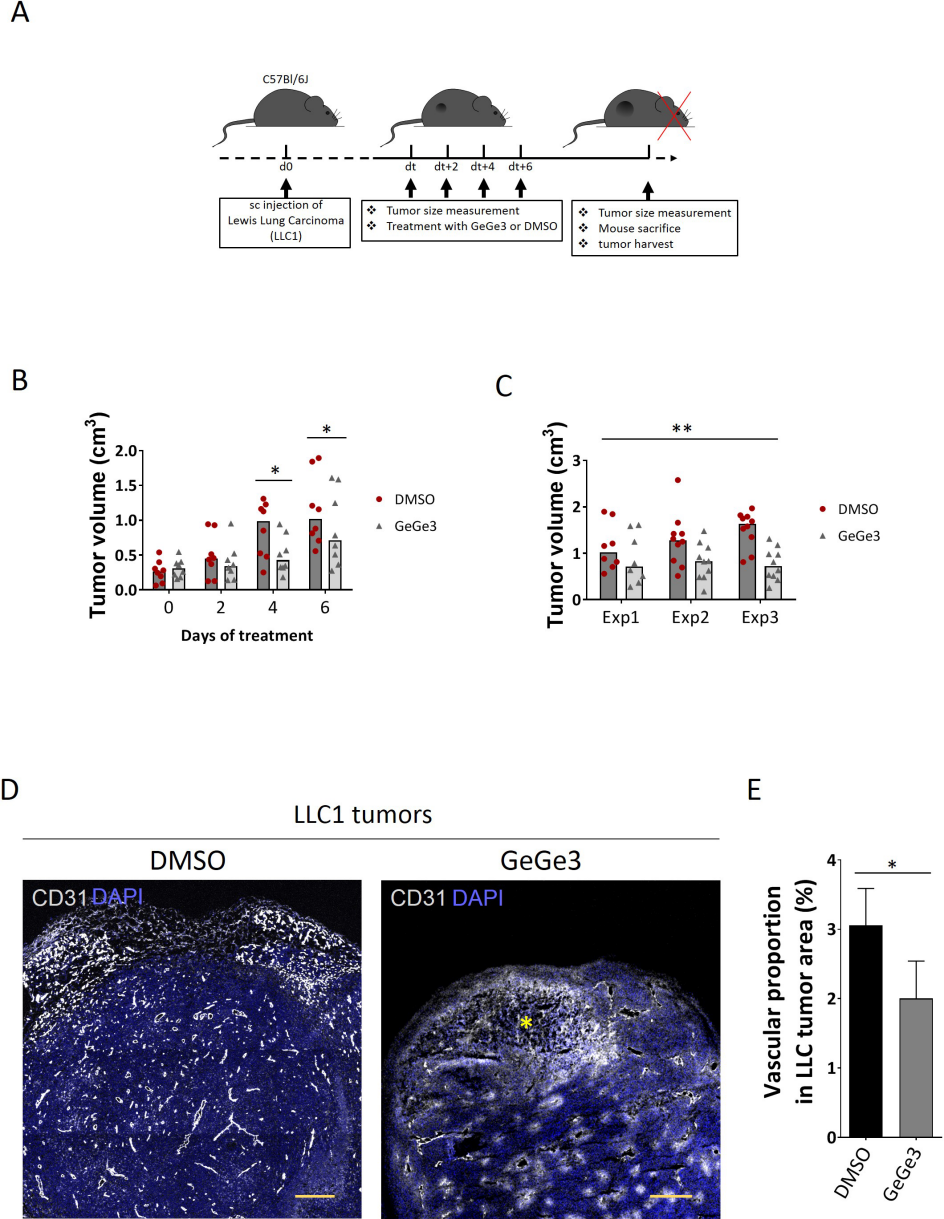
Effect of GeGe3 on LLC1 angiogenesis and tumor development **(A)** Experimental timeline for the investigation of the activity of GeGe3 on the growth of subcutaneous (s.c) Lewis Lung carcinomas (LLC1). C57Bl/6J mice were subcutaneously injected with their syngeneic LLC1 cells and tumors were left to develop up to a median of ~0.3-cm^3^ in volume before treatment with DMSO or GeGe3 (2 mg/kg) every 2 days as indicated. Tumor dimensions were measured before treatment. After 6 days, the mice were sacrificed and the tumors analyzed. **(B)** Kinetics of tumor growth in DMSO and GeGe3-treated mice. A general reduction of tumor growth was apparent 4-days after commencing treatment with GeGe3. N=10 tumors from 5 mice in each group. Data are presented as scatter dot plot with bars representing the median in groups. ^*^:p-value<0.05. **(C)** Replications of the effect of GeGe3 on LLC1 tumor growth after 6 days of treatment. N=10 tumors from 5 mice in each group. The effects of DMSO and GeGe3 are compared for three different experiments (Exp1-3), ^*^:p-value<0.05, ^***^:p-value<0.001. **(D)** Confocal imaging of tumor blood vessels with antibodies to CD31. The tumors were counterstained with DAPI. Scale bar = 300μm. A necrotic area is indicated with a yellow star. **(E)** Quantification of tumor vascular density. CD31+ areas were determined and normalized by the area of DAPI representing the entire tumor section. This proportion served as an index of vascular density in each tumor. N=6 tumors (10 sections/tumor) per group were analyzed for tumor vascularization. Data are presented as mean ± sem. ^*^:p-value<0.05.

Next we used the LLC1 tumor model to test the effect of GeGe3 and DMPK1 on angiogenesis. We reasoned this was a good model to use as the proliferation of this cancer cell type was not affected by GeGe3 treatment. C57BL/6J mice were subcutaneously injected with LLC1 cells to develop measurable tumors (Figure 7A). After 10 days of development, when the median size of the tumors reached ~0.3-cm^3^ in volume, mice were randomly assigned to either the DMSO or the GeGe3 group of treatment. Mice were then intraperitoneally injected with GeGe3 (2 mg/kg) or control DMSO and tumors were measured every two days with a caliper. After six days of treatment, mice were sacrificed to analyze tumor vasculature. GeGe3 significantly reduced tumor size by the fourth day of treatment (Figure 7B). This difference in tumor volume was maintained at day six demonstrating an inhibitory effect of GeGe3 on tumor growth. This effect of GeGe3 on tumor growth was reproduced in three experiments as shown by the comparison of tumor volumes at endpoints (Figure 7C). Interestingly, the treatment by GeGe3 at 2 mg/kg showed no change in mouse behavior compared to DMSO-treated mice. Histological analysis of mouse kidneys after 6 days of treatment did not show any morphological difference between DMSO and GeGe3 treated animals (Supplementary Figure 5A). This demonstrated that mouse treatment by GeGe3 for 6 days was not toxic at the concentration of 2 mg/kg. We next analyzed the vascular density of LLC1 tumors to investigate whether an action on tumor angiogenesis could account for the anti-tumorigenic effect of GeGe3. We used tumor sections of comparable sizes from both groups to stain blood vessels with antibodies to CD31. We imaged the tumor sections by confocal microscopy and stitched images to have a broader picture of the whole tumor (Figure 7D, 7E). Tumors from the GeGe3-treated group had decreased blood vessel density with some necrotic areas (yellow star in the GeGe3-treated section). Altogether, these results demonstrate an inhibitory effect of GeGe3 on tumor angiogenesis *in vivo* and point towards its potential as a candidate compound for the treatment of tumor angiogenesis.

### GeGe3 targets angiogenesis through DMPK1

**Figure 8:**
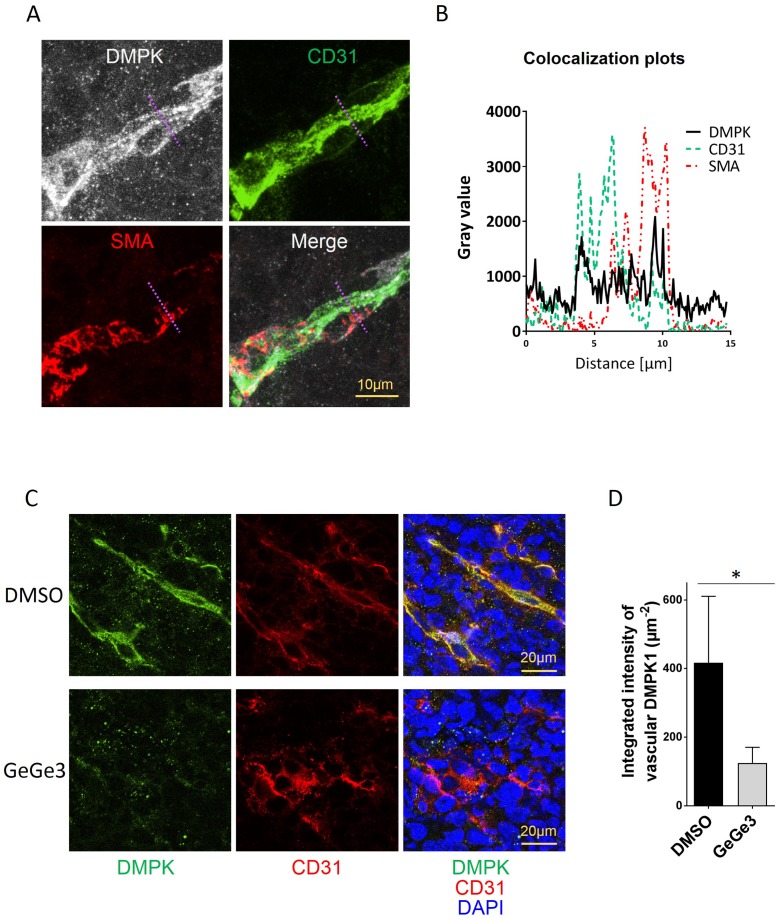
Effect of GeGe3 on DMPK expression in LLC1 vasculature **(A)** Confocal analysis of DMPK expression in endothelial cells and pericytes in LLC1 tumors. DMPK colocalized with both endothelial cells (CD31+) and pericytes (a-smooth muscle actin, SMA positive). A region of interest (R.O.I: dashed violet line) was plotted in (B). Scale bar = 10 μm **(B)** Colocalization plots of DMPK with endothelial cells (CD31+) and pericytes (SMA+) in LLC1 tumors. The highest expression levels of DMPK were found in endothelial cells and pericytes compared to tumor cells. **(C)** Effect of GeGe3-treatment on DMPK expression, analyzed by confocal microscopy. Scale bar = 20μm. **(D)** Quantification of DMPK expression in sections of LLC1 tumors of DMSO- vs GeGe3-treated mice. GeGe3-treatment decreased DMPK expression in tumor endothelial cells. N=5 tumors (10 sections analyzed/tumor) per treatment. Data are presented as mean±sd. ^*^:p-value<0.05.

Finally, we investigated the expression of DMPK in the blood vessels of LLC1 tumors. The stability of blood vessels depends not only on endothelial cells but also on mural cells including smooth muscle cells or pericytes wrapping around the endothelium [43]. We analyzed DMPK1 expression in both endothelium and mural cells of tumor blood vessels, identified by CD31 and smooth muscle cell actin (SMA) respectively. As shown in Figure 8A, DMPK expression was detected in both endothelial cells and mural cells. Indeed, colocalization of DMPK and CD31 and SMA demonstrated high DMPK levels in both endothelial cells and pericytes (Figure 8B, Supplementary Video 1). The treatment of the mice with GeGe3 reduced the DMPK protein level in blood vessels of LLC1 tumors (Figure 8C, 8D). Interestingly, mouse treatment at 2 mg/kg for 6 days did not affect DMPK expression in normal tissues such as kidneys (Supplementary Figure 5B). This result is further evidence that GeGe3 affects tumor angiogenesis by targeting DMPK activity and protein level in the tumor microenvironment but not in normal tissues as shown for kidneys.

## DISCUSSION

In this study we demonstrate that GeGe3 is a new promising antiangiogenic compound. We show this *in vitro*, where GeGe3 blocks HUVEC proliferation and tube formation. *In vivo*, we also report that GeGe3 inhibits physiological intersegmental angiogenesis in zebrafish embryos and pathological angiogenesis of tumors.

Consistent with a specific effect on angiogenesis we found that GeGe3 did not affect the proliferation of human colorectal adenocarcinoma cells or mouse Lewis Lung carcinoma cells. Together these results confirm GeGe3 as an interesting potential antiangiogenic drug. The strongest illustration of this is our demonstration that not only does GeGe3 inhibit angiogenesis but it also decreased tumor growth within 4 days of initiating treatment at 2 mg/kg (Figure 7B, 7C). At this dose, GeGe3 administration did not show noticeable toxicity as shown by the absence of morphological and behavioral differences between GeGe3 and DMSO treated mice. The used GeGe3 amount remains a low dose compared to other compounds in many *in vivo* studies. We did not test higher doses due to GeGe3 solubility limitation and constrains related to the administration mode. Higher doses of GeGe3 by other administration methods such as by gavage may be interesting to test in future experiments. Longer duration of mouse treatment with GeGe3 at 2 mg/kg may be an alternative to increase the benefit for tumor therapy and short term treatment showed remarkable effects. To further develop our novel compound, it will also be interesting to test GeGe3 on different tumor cell lines to verify its inhibitory action. It will also be interesting to investigate the general applicability of GeGe3 on different types of cancer. However, these experiments are beyond the present manuscript.

Here we also refine the molecular targets of GeGe3. Antiangiogenic pyrazole derivatives like GeGe3 that target tumor blood vessels are known to have a multitude of molecular targets. However, most attention thus far has focused on tyrosine kinases [24–30]. We recently showed that GeGe3 interferes with MAPK and PI3K signaling pathways as it alters ERK1/2 and AKT activation [37]. However, we found in our kinome analysis in this study that its actual molecular targets in endothelial cells were Aurora B, Aurora C, NEK 10, PLK2, PLK3, DMPK1 and CaMK1; none of which are classical components of MAPK and PI3K signaling. However, all these kinases are implicated in cell division and/or calcium homeostasis. By directly targeting the activity of these kinases, GeGe3 also acts indirectly on the extent of MAPK and PI3K activation, leading to a further inhibition of endothelial cell proliferation. Consistently, the downregulation of GeGe3-targeted DMPK1 led to a reduced activation of ERK1/2 by VEGF stimulation of endothelial cells. Thus, a direct action of GeGe3 on these kinases could account for its inhibitory effect on angiogenesis.

We found among GeGe3-targeted kinases that DMPK1 activation is induced by VEGF stimulation and consistently decreased by GeGe3 in HUVECs. DMPKs are serine/threonine kinases that are essential for skeletal muscle function, myocyte differentiation and synaptic plasticity of neurons [41, 42, 44]. DMPKs remained unexplored in endothelial cell physiology until this study. Herein we found that DMPK role in VEGF signaling pathways is essential for endothelial cell proliferation and migration.

A previous study has shown that DMPK interacts with and is activated by Raf-1 kinase, upstream of ERK activation in MAPK signaling pathway [45]. This might explain why VEGF induces DMPK. Indeed VEGF induces Raf-1 activation through VEGFR2, upstream of ERK induction [46]. However, it remains elusive how DMPK-ERKs crosstalk operates. This crosstalk may be related to DMPK involvement in cellular calcium homeostasis, previously described for cardiomyocytes [42]. However, we did not find any change in the CaMK1 activation status upon VEGF challenge of HUVEC during early time periods, demonstrating that this calcium-dependent kinase was not implicated in DMPK-supported MAPK signaling. Other calcium-dependent kinases may be involved. We suspect a possible involvement of the conventional calcium-dependent protein kinase C (PRKCA, PRKCB and PRKCG) isoforms in this process, as some of them were reported to be required for full activation of ERKs in MAPK signaling pathways and angiogenesis [47, 48]. The potential role of DMPK in PKC activation through modulation of calcium uptake in endothelial cells may be the missing link between DMPK and MAPK, which is to be investigated in the future. Consistently, in endothelial cells, DMPK was found to be located in structures resembling the endoplasmic reticulum, which is the cellular calcium reservoir.

In this study we showed, for the first time, that DMPK has a role in VEGF-induced activation of MAPK in endothelial cells, as well as their proliferation and migration required for normal and tumor-associated angiogenesis. We also report that the pyrazolyl-urea GeGe3 blocks tumor angiogenesis through targeting DMPK activity and protein level. The integrated effect of blocking additional kinases implicated in cell division, such as Aurora C and PLK2, may further support the efficacy of GeGe3 in inhibiting angiogenesis.

In the future it will be interesting to investigate the role of DMPK's interacting partners in endothelial cells and their roles in the physiology of these cells. Any endothelial-specific role of DMPK and partners will also provide novel alternative therapeutic targets. The development of more efficient pyrazole derivatives based on the GeGe3 structure and its interaction with DMPKs will likely bring new classes of antiangiogenic drugs to test and eventually implement in the clinic.

In summary, we report that the pyrazolyl-urea GeGe3, which is a novel blocker of MAPK and PI3K pathways in endothelial cells, can strongly inhibit physiological and tumor angiogenesis. GeGe3 appears to work by targeting kinases that are essential for cell division and calcium homeostasis. Among the direct targets of GeGe3, our study revealed DMPK as a novel player in angiogenesis. Our study thus highlights GeGe3 as a potential antitumor therapeutic to further investigate and serve as a structural base for the development of more potent antiangiogenic compounds that can target signaling pathways common to VEGF and compensatory angiogenic factors.

## MATERIALS AND METHODS

### Mice and zebrafish embryos

Six-week old male C57BL/6J mice were purchased from Charles River Laboratories (Lyon, France). In subcutaneous tumor development experiments, mice were randomly assigned to experimental groups. To study physiological angiogenesis during development, *Tg(fli1:EGFP)y1* zebrafish [38], expressing enhanced green fluorescent protein (EGFP) in vessel endothelium, were crossed with casper fish. Transgenic *Tg(fli1:EGFP)y1* fish were obtained from the Zebrafish International Resource Center at the University of Oregon. They were generated in the laboratory of Brant M. Weinstein. The casper strain was used for crossing convenience. EGFP-positive embryos, at approximately the 20-21 somite developmental stage, were incubated for 8 hours at 28.5 °C with GeGe3 (20 μM), or DMSO vehicle control, in E3 medium (5 mM NaCl, 0.17 mM KCl, 0.33 mM CaCl2 and 0.33 mM MgSO_4_). De-chorionated embryos were then imaged under anaesthesia in E3 medium supplemented with ethyl 3-aminobenzoate methanesulfonate salt (Tricaine, 150 μg/mL). Whole pictures of embryos were acquired at room temperature with a Leica MZ16FA stereomicroscope and a Leica DFC340FX digital camera, using Leica LAF software. For vessel analysis, embryos were fixed post-treatment with 4% paraformaldehyde (PFA) overnight and intersegmental vessels imaged with a Nikon Ar1 reverted confocal microscope. Images were processed using Imaris 8.3 software (Bitplane) and vessel length quantified using ImageJ software. All procedures were performed in agreement with the Institutional Ethics Committee of Animal Care in Geneva and the Swiss Cantonal Veterinary Office.

### Cell culture

We used human umbilical vein endothelial cells (HUVEC) isolated in our laboratory in agreement with the institutional ethics committee. The cell lines of human colorectal adenocarcinoma DLD1 and mouse Lewis Lung carcinoma (LLC)1 were purchased from American Type Culture Collection (ATCC).

#### HUVEC culture

cells were seeded in plates or dishes pre-coated with 0.2% of gelatin and Collagen G at 0.1 mg/ml in PBS. The cells were cultured in M199 medium (GIBCO) supplemented with 10% fetal calf serum, 1% Endothelial Cell Growth Supplement (EmdMillipore), 0.1 mg/ml of heparin sodium, 0.1 μM of hydrocortisone (Sigma), 1% antibiotics-glutamine mixture, 0.1% of vitamin C and incubated at 37°C under a humidified atmosphere containing 5% CO2.

#### Cancer cell culture

DMEM medium (GIBCO) was used for the murine Lewis lung carcinoma cell line LLC1 and RPMI medium (GIBCO) for the human colorectal cancer cell line DLD1, both supplemented with 10% fetal calf serum and incubated at 37°C under a humidified atmosphere containing 5% CO_2_, as recommended by ATCC.

### Compound preparation

The Ethyl 1-(2-hydroxypentyl)-5-(3-(3-(trifluoro methyl)phenyl)ureido)-1H-pyrazole-4-carboxylate (named GeGe3, see Supplementary Figure 1) was from a library of pyrazolyl-ureas and imidazopyrazoles (27 compounds) that we synthesized [37]. In this study, GeGe3 was dissolved in DMSO and diluted in the culture medium at the specified concentrations. Thus DMSO was used as control condition in all experiments. GeGe3 was generally used at 20μM unless stated.

### Reagents

The 5-chloromethylfluorescein diacetate (CMFDA) fluorescent dye was used to stain HUVEC in migration assays (Themo fisher Scientific). The 5-ethynyl-2'-deoxyuridine (EdU) assay was performed with the Click-iT^®^ kit (Themo fisher Scientific). For *in vitro* angiogenesis we used Geltrex™ LDEV-Free Reduced Growth Factor Basement Membrane Matrix (Thermo Fisher Scientific). We used 4',6-diamidino-2-phenylindole (DAPI) for nuclear staining and propidium iodide (PI) for testing cell membrane integrity (both from Thermo Fisher Scientific). For screening the activity of the serine/threonine kinome of HUVECs, we used PamChip^®^ arrays on a PamStation12^®^ instrument (Pamgene). Our list of antibodies and their working concentrations can be found in the Supplementary Materials.

### Proliferation assay

Cells were seeded in a 96-well plate at 1000 cells/well and cultured for 4-Hrs. After this incubation cells were treated with VEGF at 50 ng/ml (only HUVEC), EdU (5-ethynyl-2'-deoxyuridine) at 2.5 μM and GeGe3 at20 μM, overnight. In a control condition, DMSO was used. Conditions without EdU served as negative control for the specific detection of EdU in the nucleus. After 24-Hrs the cells were washed with PBS and then fixed with 4% paraformaldehyde (PFA) for 10 min at 4°C and permeabilised with 0.2% Triton-X-100 for 5 min at room temperature. EdU was stained with the Click-iT^®^ reaction cocktail as described by the manufacturer (ThermoFisher) and cell nucleus was stained with DAPI for 5 min at room temperature. The cells were photographed with an ImageXpress microscope. Cells were counted with the count nuclei tool within the ImageXpress software and the data were expressed as percentage of proliferating cells with respect to the total cell number.

### Angiogenesis assay

A 96-well μ-plate (IBIDI) was coated with Geltrex™ diluted in fresh M199 medium (1:1 ratio) and incubated for 10 min at 4°C and for 20 min at 37°C in a humidified atmosphere containing 5% CO_2_. After polymerization, HUVEC were re-suspended in complete culture medium containing GeGe3 at 20 μM and VEGF at 50 ng/ml and seeded at 10.000 cells/well. Cells were photographed with ImageXpress for 10-Hrs. The images were analyzed with ImageJ using the Angiogenesis analyzer toolset. Data were presented as the total length of the tubing segments and the network stability index, which is the ratio of total segment length and the number of isolated segments.

### Testing GeGe3 activity on LLC1 tumor growth *in vivo*

C57BL/6 mice were subcutaneously injected with syngeneic LLC1 carcinoma cells at 10^6^ cells/injection at two different sites. After 10 days of tumor development, the mice were either treated with GeGe3 (2 mg/kg) or DMSO by intraperitoneal injection. The tumor size was measured with a caliper every 2 days and the treatments delivered after the measurement. After one week of treatment, the mice were euthanized and the tumors recovered for immunohistochemistry.

### Immunofluorescence and immunohistochemistry

Confluent or sparse HUVEC were fixed with 4% PFA for 10 min at 4°C and permeabilized with 0.2% triton X-100 for 5 min. Cell were blocked with blocking buffer made of PBS containing 2% BSA, 20% FCS, 5% normal donkey serum and 0.1% tween20. Primary antibodies or isotype controls were applied for 1-Hr and adequate secondary antibodies were applied with DAPI (1/100). The samples were mounted with Hardset mounting medium (Vectashield) and imaged with a super-resolution confocal microscope LSM800 (Zeiss).

For immunohistochemistry of tumors, pieces of tumors were embedded in Tissue-Tek^®^ O.C.T compound Sakura® (VWR) in liquid nitrogen. Ten sections of 8-μm thick were used per tumor for fixation with 4% PFA, permeabilization with 0.2% triton X-100, blocking and staining as described above. Sections were imaged with Nikon Ar1 confocal microscope (Nikon) and images were stitched to capture the entire cross-section of the tumors. CD31+ areas were determined and normalized by the area of DAPI representing the entire tumor section. This proportion served as an index of vascular density in each tumor. For DMPK colocalization with CD31 and a-SMA in LLC1 tumors, z-stacks of the sections were acquired and the 3D reconstruction was performed with Imaris 8.3 software (Bitplane). Colocalization was analyzed with a region of interest (R.O.I) defined with a line as indicated. This was performed with ImageJ software.

### Exploration of MAPK and PI3K signaling pathways

Confluent HUVEC were starved for at least 4-Hrs with fresh M199 medium before stimulation. HUVEC were pre-incubated with DMSO or GeGe3 at 20 μM for 10 minutes and then VEGF (50 ng/ml) was added for 5 to 20 minutes unless otherwise indicated in the figure legends. Cells were washed and lysed for protein extraction. Protein concentration was determined with the MicroBCA™ Protein Assay Kit (Thermo Scientific). Western blotting was performed as described previously. Equal amounts of proteins (30 μg) were subjected to a gel electrophoresis and transferred to nitrocellulose blotting membranes. Membranes were blocked with a blocking buffer made of 1xPBS containing 5% not-fat dry milk and 0.05% of Tween20. Membranes were incubated overnight at 4°C with primary antibodies diluted in the blocking buffer. Membranes were washed three times for 5 minutes with PBS-Tween20 0.05% and incubated at room temperature for 1 hour with secondary antibodies. Membranes were washed three times for 10 minutes and the signals detected using the enhanced chemioluminescence system (Advansta, WesternBright™ Sirius). Protein band intensities were quantified with ImageJ. Tubulin intensity was used to ensure equal protein loading. The results were expressed relative to the condition of treatment with VEGF and DMSO serving as control in the each blot, set at 100%. Data are representative of at least two independent experiments.

### Survey of GeGe3 targeted kinases in HUVEC

Protein extract of VEGF-stimulated HUVEC was used as the source of kinases to run the PamChip kinase assay (Pamgene) according to the manufacturer's protocol. Sample incubation, detection, and analysis were performed on a PamStation12 according to the manufacturer's instructions. Quantification and statistical analysis was performed by PamGene International BV using BioNavigator software. Details can be found in Supplementary Materials.

### DMPK downregulation by siRNA

HUVECs were transfected with a mix of 3 unique 27mer siRNAs (rGrGrCrGrArGrArCrCrUrArUrGrGrCrArArGrArUrCGTC, rArGrArUrCrArUrGrArArCrArArGrUrGrGrGrArCrArUrGCT, rGrCrArCrGrGrArCrArArCrCrArGrArArCrUrUrCrGrCrCAG) duplexes that all target DMPK (Origene). A universal scrambled negative control siRNA duplex was used as control. The transfection was performed with Lipofectamine^®^ 2000 (Thermo Fisher Scientific) according to the manufacturer instructions. 72 hours after transfection, DMPK expression level was analyzed by western blotting.

### Analysis of DMPK expression by quantitative polymerase chain reaction (qPCR)

HUVECs were treated with GeGe3 for 5 hours before washing and RNA extraction with an RNA isolation kit (Macherey-Nagel). Total mRNA was reverse transcribed with SuperScript II Reverse Transcriptase (Thermo Fisher Scientific) and used for qPCR with the specific primers listed below with a StepOnePlus Real Time PCR machine (Applied Biosystems) Relative quantification was performed with DataAssist3 analysis software (Thermo Fisher Scientific). Gene expression was normalized to *GAPDH* and *BACT*.

The following qPCR primers were used in this study.

**Table:** 

Name	Forward	Reverse
*Hu DMPK*	TGGCGGAGATTGTCATGGC	GGATGTTGTCGGGTTTGATGTC
*hu BACT*	GCAAAGACCTGTACGCCAAC	CTAGAAGCATTTGCGGTGGA
*hu GAPDH*	AGAAGGCTGGGGCTCATTTG	AGGGGCCATCCACAGTCTTC

### Statistics and data presentation

All data are presented as mean±standard error of mean (sem) and by scatter dot plot with mean. DMSO and GeGe3 groups from independent experiments were compared by unpaired Student's t-test or Mann-Whitney non-parametric test, as indicated, using GraphPad Prism 7.

Please see Supplementary Materials for the antibody list, propidium iodide uptake test and details of the serine/threonine kinase arrays.

## SUPPLEMENTARY MATERIALS FIGURES AND TABLE





## Abbreviations

Ang: angiopoietin
bFGF: basic ibroblast growth factor
CaMK1: Calcium/calmodulin-dependent protein kinase type 1
DAPI: 4',6-diamidino-2-phénylindole
DMPK: Dystrophia Myotonica Protein Kinases
EdU: 5-Ethynyl-2'-deoxyuridine
EGFP: enhanced green fluorescent protein
ERK: extracellular response kinases
GeGe3: ethyl 1-(2-hydroxypentyl)-5-(3-(3-(trifluoromethyl)phenyl)ureido)-1H-pyrazole-4-carboxylate
HUVECs: human umbilical vein endothelial cells
IL: interleukin
LLC: Lewis lung carcinoma
MAPK: mitogen-activated protein kinases
PDGFR: Platelet-derived growth factor receptors
PI3K: phosphoinositide 3-kinase
PKB/AKT: protein kinase B
PLGF: placental growth factors
PLK: polo like kinases
SMA: smooth muscle cell actin
VEGF: vascular endothelial growth factor
VEGFR: VEGF receptors
