# Supplementary material for: The pyrazolyl-urea GeGe3 inhibits tumor angiogenesis and reveals dystrophia myotonica protein kinase (DMPK)1 as a novel angiogenesis target
Source: Oncotarget. 2017 Nov 21;8(64):108195–212. doi: 10.18632/oncotarget.22598 (PMC5746136; doi:10.18632/oncotarget.22598)
Supplement: Supplementary file 1 [file oncotarget-08-108195-s001.pdf]

The pyrazolyl-urea GeGe3 inhibits tumor angiogenesis and reveals dystrophia myotonica protein kinase (DMPK)1 as a novel angiogenesis target

SUPPLEMENTARY MATERIALS

Antibodies

The following antibodies were used in this study for western blotting, immunoprecipitation or for immunofluorescence imaging:

Names		Manufacturer	Origin	Reactivity
Primary antibodies				
phospho-ERK1/2 (pERK1/2)	WB:1/1000	Cell signaling	Rabbit	Human
ERK 1/2	WB:1/1000	Cell signaling	Mouse	Human
phospho-p38MAPK (pp38MAPK)	WB:1/1000	Cell signaling	Rabbit	Human
p38MAPK	WB/1/1000	Cell signaling	Rabbit	Human
phospho-AKT-T308 (pAKT)	WB:1/4000	Cell signaling	Rabbit	Human
phospho-AKT-S473 (pAKT)	WB:1/2000	Cell signaling	Rabbit	Human
AKT	WB:1/2000	Cell signaling	Rabbit	Human
α-tubuline	WB:1/4000	Millipore	Mouse	Human
Aurora C	WB:1/500	Genetex	Rabbit	Human
Polo-like kinase 2 (PLK2)	WB:1/500	Genetex	Rabbit	Human
DMPK	WB:1/500, IF:1/100	Genetex	Rabbit	Human, mouse
DMPK	WB:1/500	Biotechne	Mouse	Human
Phospho- threonine PTR-8	IP:5µg/ml	SigmaAldrich	Mouse	Human
Phospho-serine PSR-45	IP:5µg/ml	SigmaAldrich	Mouse	Human
Phospho-threonine 42H4 IgM	WB:1/1000	Cell signaling	Mouse	Human
VE-cadherin-FITC, REA199	IF:1/100	Miltenyi Biotech	Human	Human
REA Control (S)-FITC	IF:1/100	Miltenyi Biotech	Human	none
CD31-Alexa Fluor 647, clone 390	IF:1/200	Biolegend	Rat	Mouse
a-SMA-biotin, 1A4, IgG2a	IF:1/200	Skalli et al.	Mouse	Human, mouse
Actin	WB:1/5000	Millipore	Mouse	Human
Phospho-CaMK1(Thr177)	WB:1/1000	Thermofisher	Rabbit	Human
CaMK1 (H-8)	WB:1/1000	Santa Cruz BT	Mouse	Human
Secondary antibodies				
Anti-rabbit-HRP	WB:1/10000	Jackson Imm.	Goat	Rabbit IgG
Anti-mouse-HRP	WB:1/3000	Jackson Imm.	Goat	Mouse IgG
Anti-mouse IgM-HRP	WB:1/3000	Jackson Imm.	Donkey	Mouse IgM
Anti-rabbit-AF488	IF:1/500	Jackson Imm.	Donkey	Rabbit IgG
Anti-rabbit-AF594	IF:1/500	Jackson Imm.	Donkey	Rabbit IgG
Anti-mouse IgG2a-AF594	IF:1/500	Jackson Imm.	Donkey	Mouse IgG2a

PI cytotoxicity test by flow cytometry

HUVEC were seeded in 24-well plates at 30,000 cells/well and cultured overnight. The next day cells were incubated with both VEGF (50 ng/ml) and GeGe3 (20 µM) for 24-Hrs. Floating cells contained in culture supernatant were collected as well as adherent cells after detachment by trypsin treatment. Cells were centrifuged and re-suspended in PBS containing EDTA (2 mM) and BSA (0.5%). Propidium iodide (PI) at 1 µg/ml was added to the cells just before flow cytometry acquisition with a BD Accury™ C6. Data were analyzed with the BD Accury C6 Software and reported as percentage of dead cells with respect to the total cell number.

Serine/threonine kinase array assays

Confluent and starved HUVEC were stimulated with VEGF at 50 ng/ml for 10 min. Cells were lysed with the M-PER mammalian extraction buffer (Thermo Scientific) containing 1:50 Halt phosphatase inhibitor cocktail (Thermo Scientific) and 1:50 Halt protease inhibitor cocktail, EDTA-free (Thermo Scientific). The lysates were sonicated and centrifuged at 12000 g for 15 minutes to remove all debris. The supernatant was aliquoted, snap-frozen in liquid nitrogen, and stored at –80°C until further processing. Protein concentration was determined with the MicroBCA™ Protein Assay Kit (Thermofisher).

All reagents and PamChip kinase arrays used in PamGene runs were purchased from PamGene International B.V. Sample incubation, detection, and analysis were performed in a PamStation12 according to the manufacturer's instructions. Prior to incubation with the kinase reaction mix, the arrays were blocked with 2% BSA in water for 30 cycles to prevent non-specific binding and washed 3 times with protein kinase (PK) assay buffer. Kinase reaction was performed for 30 min with 1 µg of total protein extract, 20 µM of GeGe3 or DMSO, 400 µM of ATP, PK buffer, BSA, STK antibody mix in 40 µL final volume/array. Phosphorylated peptides were detected with an anti-rabbit–FITC antibodies that recognizes a pool of anti–phospho serine/threonine antibodies, in a 1 hour incubation following the PamGene STK workflow protocol. The experiment was performed in triplicate. The images obtained from the phosphorylated arrays were quantified using the BioNavigator software (PamGene International BV).

Histochemistry of treated mouse kidney

Mice were treated with either DMSO or GeGe3 (2 mg/kg) every two days for a total of six days before sacrifice. Mouse kidneys were harvested, dehydrated and embedded in paraffin for cross-section. Cross-sections (10 per organ) of 3-µm of diameter were stained with hematoxylin and eosin as previously described in Sidibe et al [1]. Stained cross-sections were imaged by using the slide scanner Axio Scan.Z1 (Zeiss). High resolution images were stitched to have global overview of the whole organ.

REFERENCE

1. Sidibé A, Polena H, Pernet-Gallay K, Razanajatovo J, Mannic T, Chaumontel N, Bama S, Maréchal I, Huber P, Gulino-Debrac D, Bouillet L, Vilgrain I. VE-cadherin Y685F knock-in mouse is sensitive to vascular permeability in recurrent angiogenic organs. Am J Physiol Heart Circ Physiol. 2014; 307:H455-63. https://doi.org/10.1152/ajpheart.00774.2013.

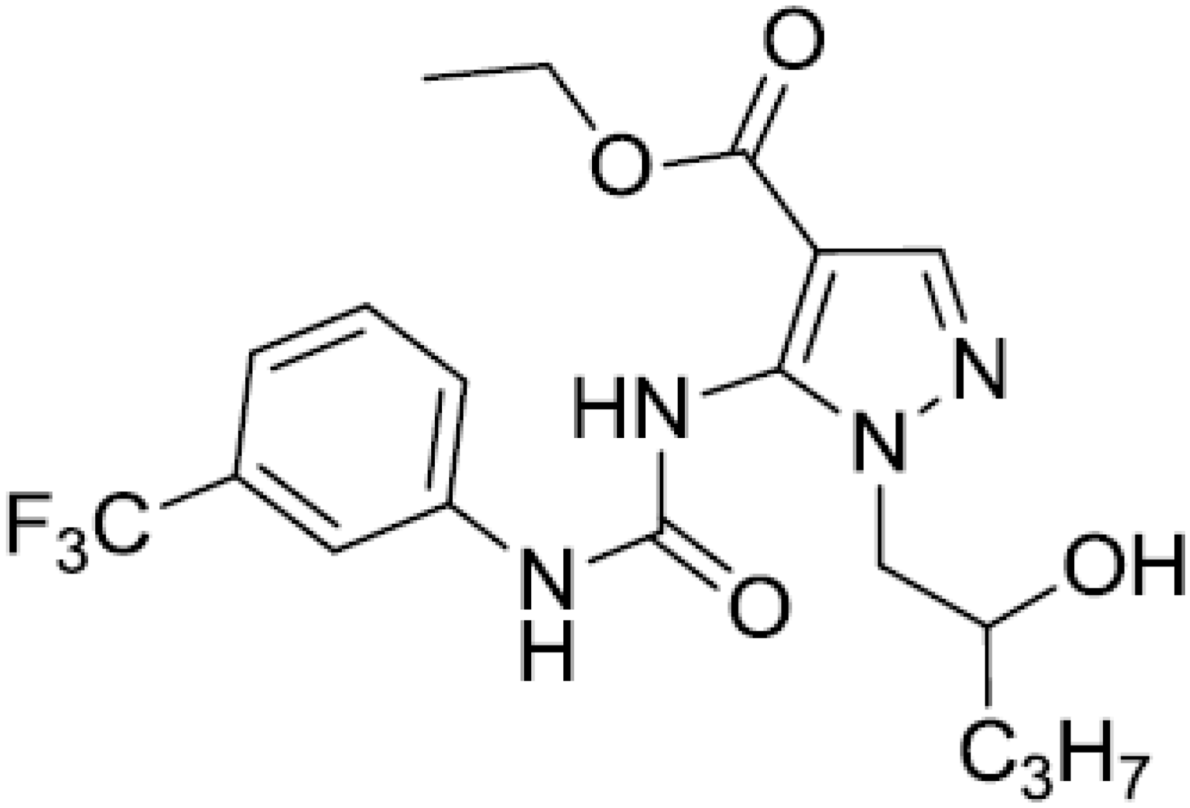

Supplementary Figure 1: Chemical structure of the pyrazolyl-urea GeGe3. GeGe3 is the Ethyl 1-(2-hydroxypentyl)-5-(3-(3-(trifluoromethyl)phenyl)ureido)-1*H*-pyrazole-4-carboxylate.

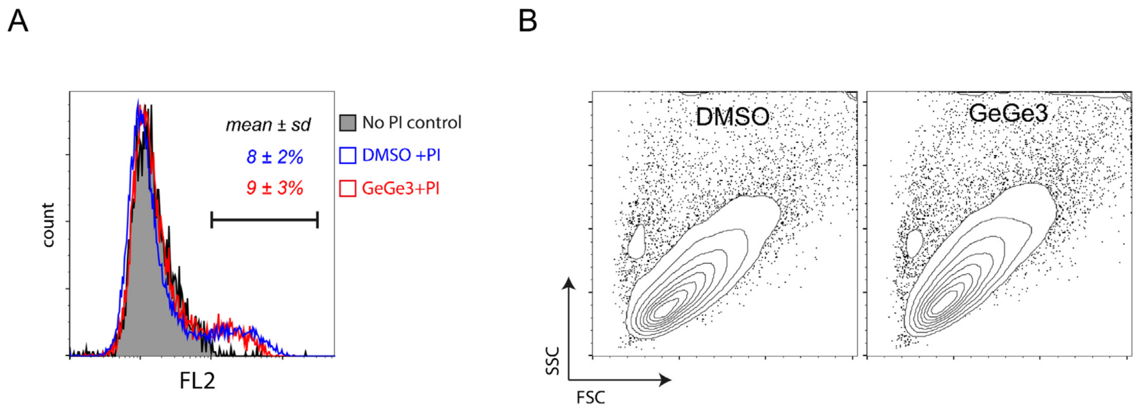

Supplementary Figure 2: Effect of GeGe3 on endothelial cell viability. (A) Analysis of the membrane integrity of HUVEC by flow cytometry. HUVEC were cultured with DMSO or GeGe3 for 24-Hrs. Floating and adherent cells were harvested and propidium iodide (PI) was added before acquisition by flow cytometry. Untreated HUVECs without PI were used as negative control. PI+ cells were quantified as apoptotic cells and used as an indicator of the compound toxicity. No difference was noticed between GeGe3- and DMSO-treated cells. **(B)** Morphology of HUVEC determined by the forward (FSC) and side (SSC) scatters by flow cytometry. HUVEC morphology was similar between DMSO and GeGe3 treatment.

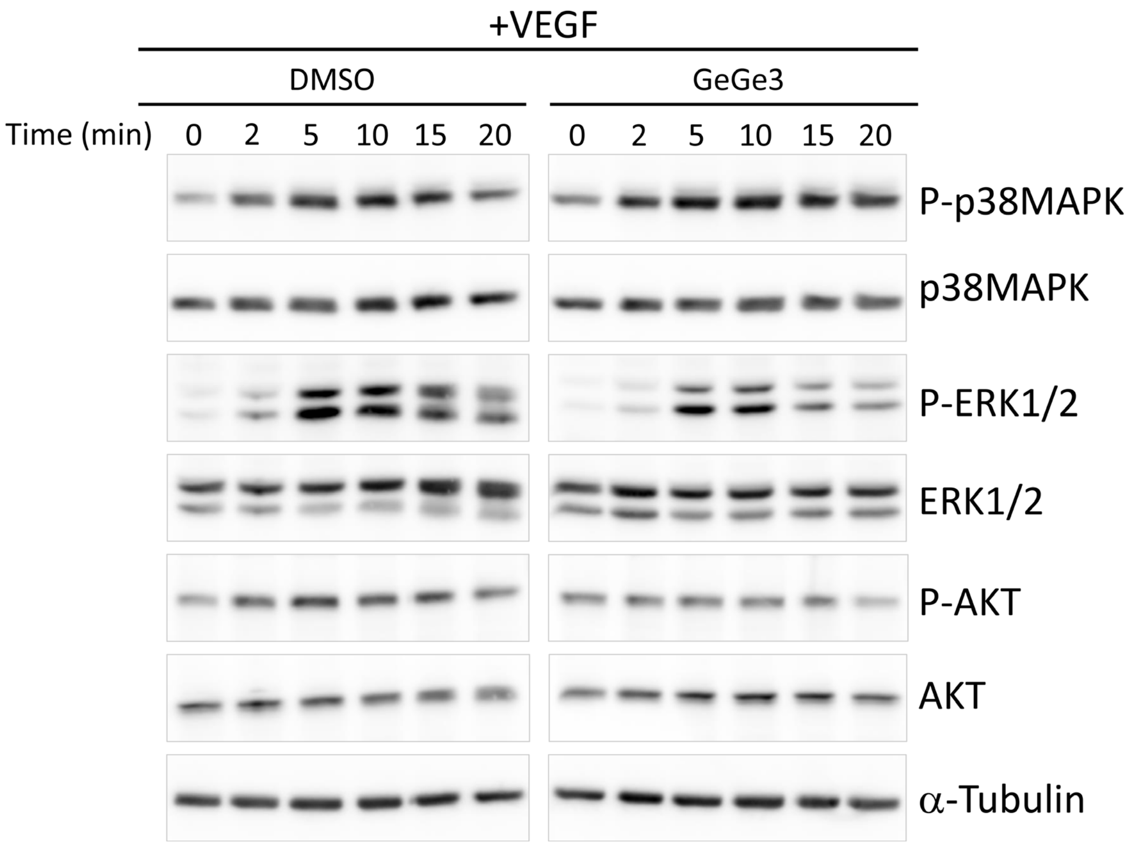

Supplementary Figure 3: Effect of VEGF and GeGe3 treatment on p38MAPK, ERK and AKT phosphorylation and protein levels. Kinetic effect of GeGe3 on VEGF-induced activation of p38MAPK, ERK and AKT. Confluent HUVECs were starved for at least 4 h. The cells were then incubated for 10-min with fresh medium containing GeGe3 or control DMSO and then VEGF (50 ng/ml) was added for the different time periods shown. Finally protein extracts were analyzed for phosphorylation of p38MAPK, ERK1/2 and AKT by Western blotting. The total protein levels of p38MAPK, ERK and AKT were analyzed as well as α-tubulin levels for protein loading controls. Whereas changes in kinases phosphorylations over time were observed, no change in kinase total protein levels was noticed. Data are representative of 3 independent kinetic experiments.

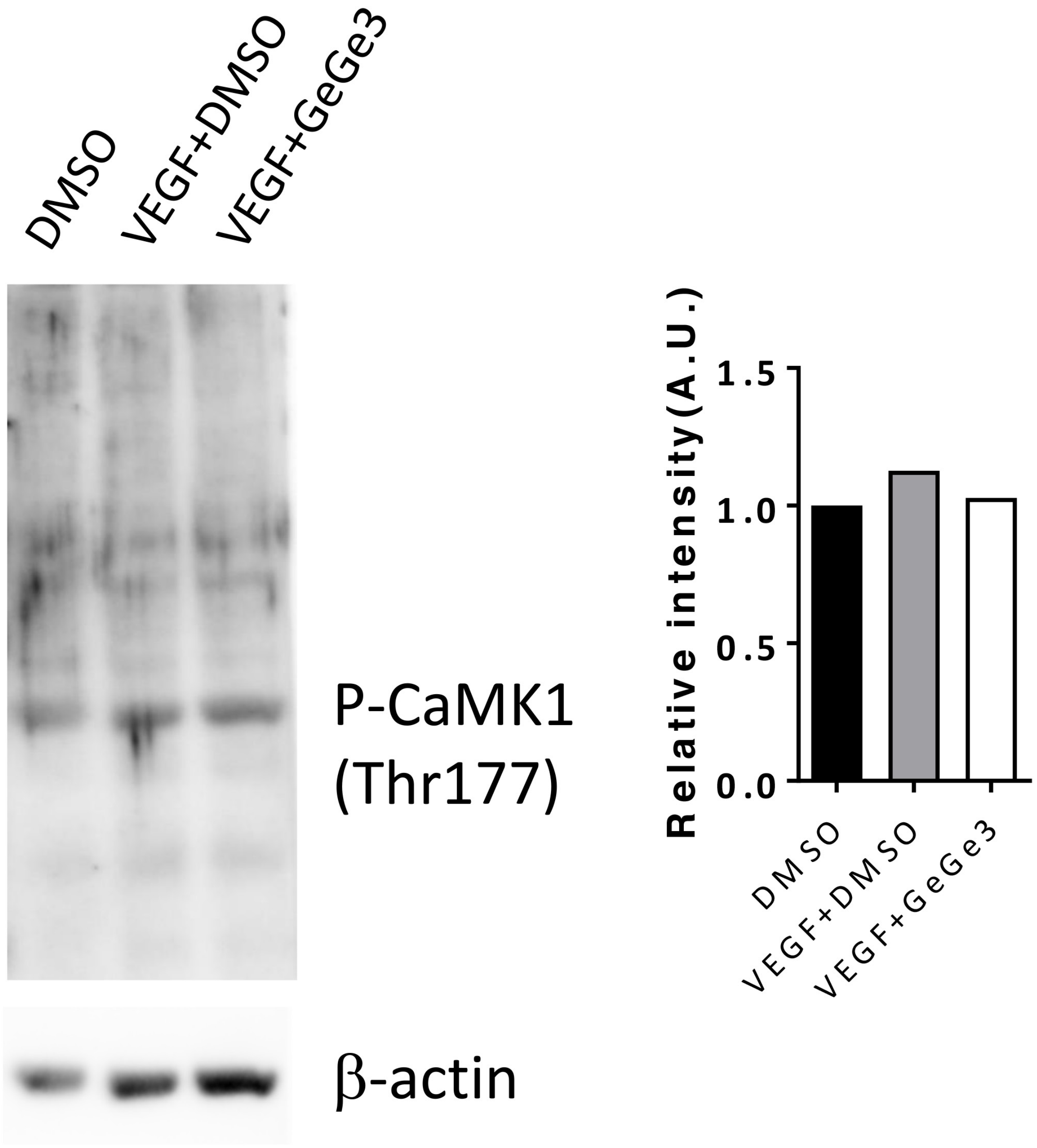

Supplementary Figure 4: Effect of VEGF and GeGe3 treatment on CaMK1 phosphorylation. Confluent HUVECs were starved for at least 4 hours. Next the cells were incubated for 10 min with fresh medium containing GeGe3 or control DMSO and then VEGF (50 ng/ml) was added for 5-min before protein extraction. Phosphorylated CaMK1 (P-CaMK1) was analyzed with a phophosite specific antibody targeting phospho-Threonine 177 of CaMK1. P-CaMK1 was normalized on β-actin levels used as loading control. Band intensity level is indicative of the phosphorylated form of CaMK1.

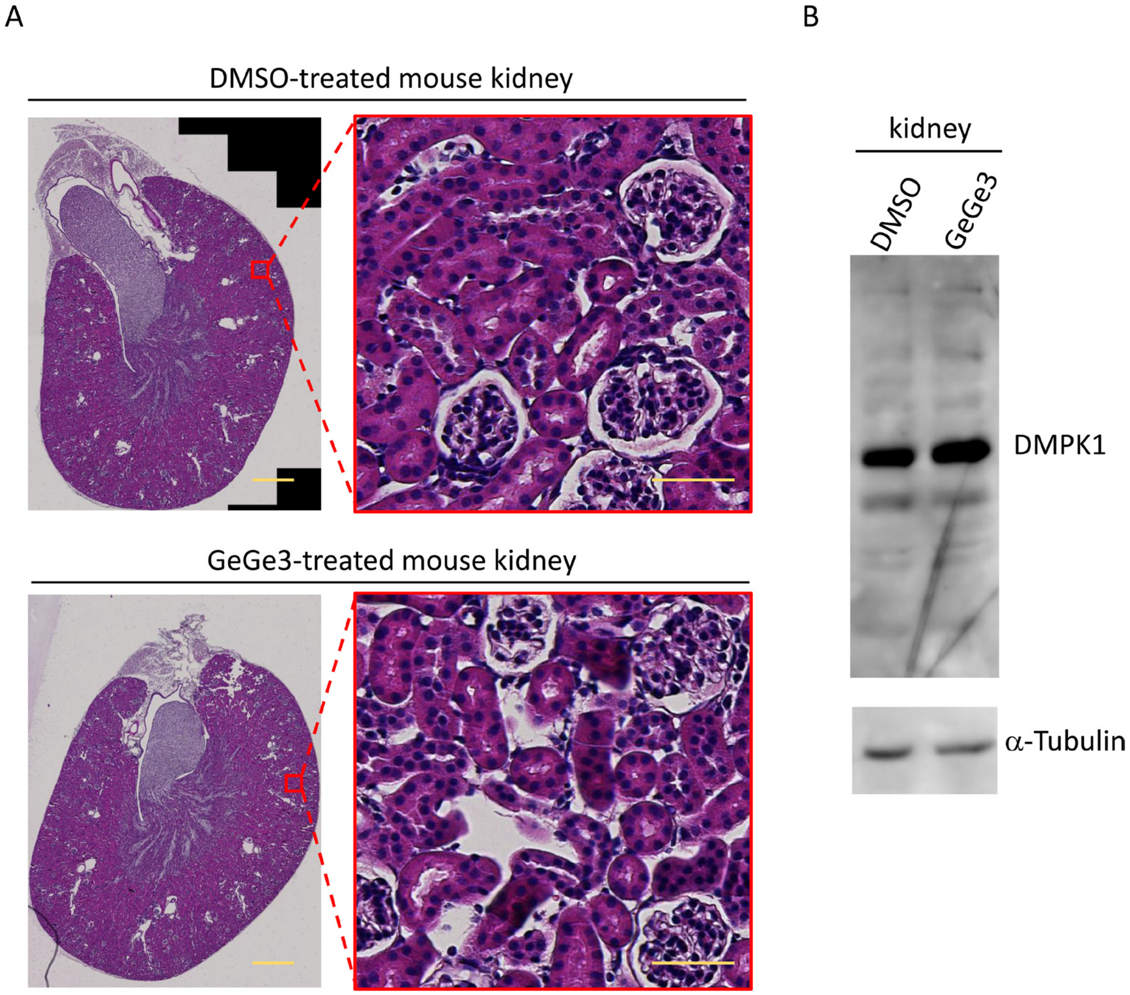

Supplementary Figure 5: Effect of GeGe3 on DMPK expression and eventual toxicity in mouse kidney. (A) Morphological analysis of GeGe3 effect on mouse kidney. Kidneys from mice treated with DMSO or GeGe3 (2 mg/kg) for 6 days were embedded in paraffin and cross-sections were stained with hematoxin/eosin to stain nuclei and cytoplasm respectively. Sections were pictured with the slide scanner Axio Scan.Z1 (Zeiss) and images were stitched. The section overviews on the left (scale bar: 500 µm) show no morphological difference between DMSO and GeGe3 treated kidneys. High magnification on the right (scale bar: 50 µm) of red section area on the left show no difference in nephron morphology. Data are representative of 10 sections per organs from 6 mice per group. **(B)** Analysis of DMPK1 expression in kidney of mice treated with DMSO and GeGe3. Tubulin expression indicates protein loading. Expression of DMPK1 in kidney was not affected by GeGe3 treatment.

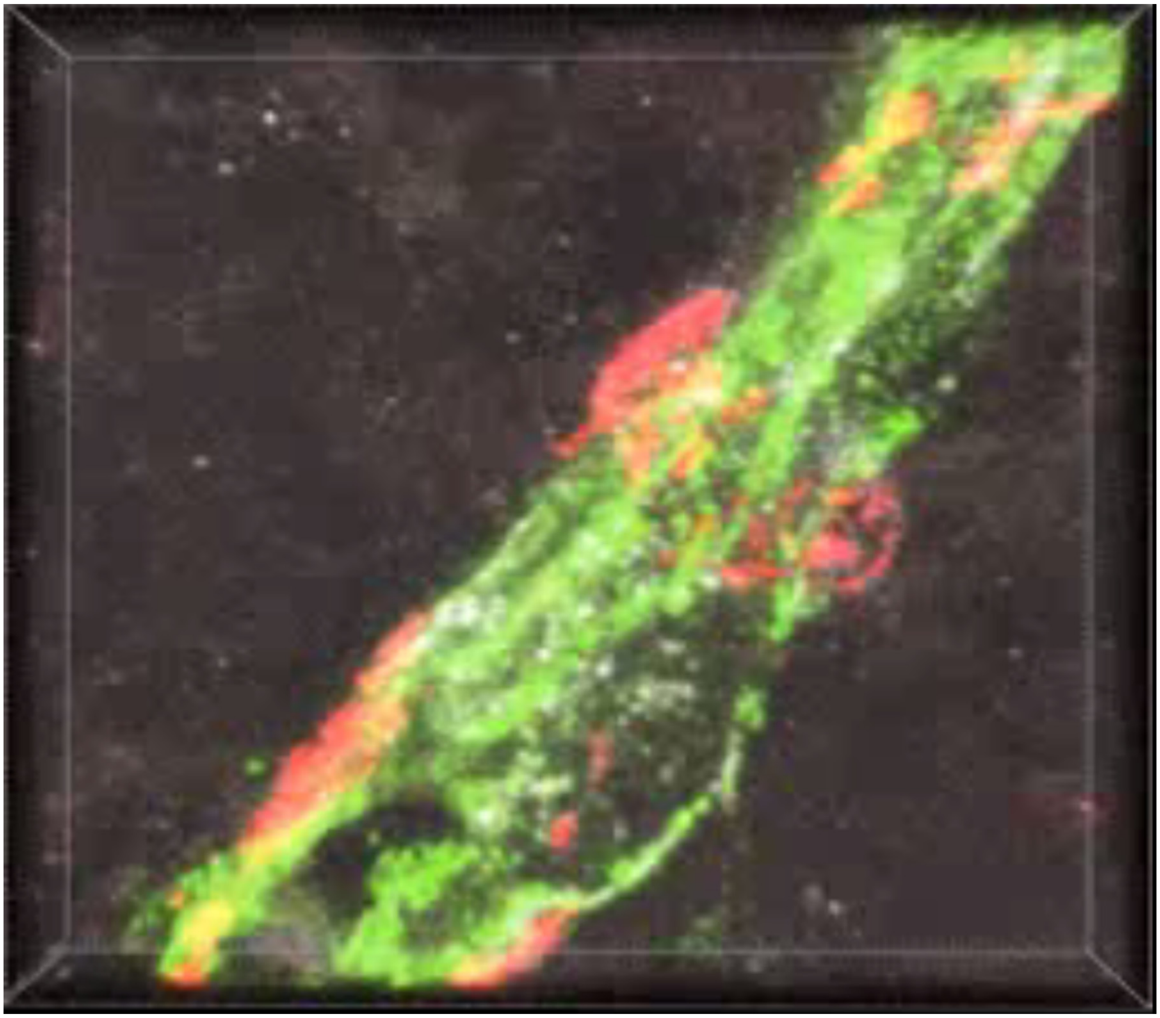

Supplementary Video 1: DMPK expression in LLC1 vasculature. Gray: DMPK, Green: CD31 (endothelial cells), Red: SMA (mural cells), Blue: DAPI (Nuclei).

See Supplementary Video 1
